# Time-restricted feeding improves adaptation to chronically alternating light-dark cycles

**DOI:** 10.1038/s41598-019-44398-7

**Published:** 2019-05-27

**Authors:** Maaike Schilperoort, Rosa van den Berg, Martijn E. T. Dollé, Conny T. M. van Oostrom, Karina Wagner, Lauren L. Tambyrajah, Paul Wackers, Tom Deboer, Gerben Hulsegge, Karin I. Proper, Harry van Steeg, Till Roenneberg, Nienke R. Biermasz, Patrick C. N. Rensen, Sander Kooijman, Linda W. M. van Kerkhof

**Affiliations:** 1 Department of Medicine, Division of Endocrinology & Einthoven Laboratory for Experimental Vascular Medicine, Leiden University Medical Center, Leiden, The Netherlands; 2 Centre for Health Protection, National Institute for Public Health and the Environment (RIVM), Bilthoven, The Netherlands; 3 Department of Molecular Medicine, University of Texas Health Science Center at San Antonio, San Antonio, TX USA; 4 Laboratory for Neurophysiology, Department of Molecular Cell Biology, Leiden University Medical Center, Leiden, The Netherlands; 5 Department of Public and Occupational Health, Amsterdam Public Health research institute, Amsterdam, UMC The Netherlands; 6 Centre for Nutrition, Prevention and Health Services, National Institute for Public Health and the Environment (RIVM), Bilthoven, The Netherlands; 7 Institute for Medical Psychology, LMU Munich, Germany; 8 Department of Human Genetics, Leiden University Medical Center, Leiden, The Netherlands

**Keywords:** Feeding behaviour, Metabolic diseases, Oscillators

## Abstract

Disturbance of the circadian clock has been associated with increased risk of cardio-metabolic disorders. Previous studies showed that optimal timing of food intake can improve metabolic health. We hypothesized that time-restricted feeding could be a strategy to minimize long term adverse metabolic health effects of shift work and jetlag. In this study, we exposed female FVB mice to weekly alternating light-dark cycles (*i*.*e*. 12 h shifts) combined with *ad libitum* feeding, dark phase feeding or feeding at a fixed clock time, in the original dark phase. In contrast to our expectations, long-term disturbance of the circadian clock had only modest effects on metabolic parameters. Mice fed at a fixed time showed a delayed adaptation compared to *ad libitum* fed animals, in terms of the similarity in 24 h rhythm of core body temperature, in weeks when food was only available in the light phase. This was accompanied by increased plasma triglyceride levels and decreased energy expenditure, indicating a less favorable metabolic state. On the other hand, dark phase feeding accelerated adaptation of core body temperature and activity rhythms, however, did not improve the metabolic state of animals compared to *ad libitum* feeding. Taken together, restricting food intake to the active dark phase enhanced adaptation to shifts in the light-dark schedule, without significantly affecting metabolic parameters.

## Introduction

Within the past decade, animal studies and epidemiologic studies have associated chronic circadian rhythm disturbance, as occurs in shift work and jetlag, with sleep problems and increased risk of cardio-metabolic disorders^1–8^. Our 24/7 economy requires people to work at irregular times. As a consequence, increasing numbers of workers are involved in atypical working schedules. Recent surveys in Europe estimate that approximately 19% of the workers in the European Union (EU) work regularly at night and 17% are involved in shift work with permanent or rotating shifts^9^. Working around the clock strains and disturbs the tightly regulated biological clock, thereby increasing the risk of poor cardio-metabolic health.

It is unlikely that the number of workers involved in atypical working schedules will decrease in the near future. Therefore, it is important that evidence-based interventions are developed to provide shift workers with tools to minimize health risks. Previous studies have shown that the timing of food intake has important consequences^10–18^. For example, Arble *et al*^14^. showed that restricting access of high-fat diet to the resting phase results in increased weight gain in mice, relative to restricting access to the active phase. Moreover, providing food during only the active phase of animals is associated with protective effects on metabolic health^10,15^. Hence, time-restricted feeding could prove an interesting strategy to minimize health risks of shift work. However, previous studies mainly investigated relatively short-term effects, whereas the precise role of food timing during chronic disturbance of circadian rhythms remains unclear.

In this study, we investigated the effect of time-restricted feeding on adaptation to weekly alternating light-dark cycles and on metabolic parameters in wild type female FVB mice. In addition, we studied the effect of food timing on predicted sleep duration in mice, as sleep might be an important intermediate factor in the effects of circadian disturbance on metabolic health.

## Methods

### Animals

This study was performed in female FVB mice (Harlan), in order to compare the outcomes of this study to our previous findings in *p53*^*R270H/+*^ *WAPCre* female FVB mice^19^. Mice were housed in cages containing wood fibre bedding (LIGNOCEL BK8/15, JRS) under controlled conditions, namely 20–21 °C, 55–65% relative humidity, and 12:12 light-dark cycle with lights on at 10.00 am, *i*.*e*. Zeitgeber Time (ZT) 0. Before starting the experimental procedure with changing light schedules and food availability (see description below; at 10 weeks of age), chow (No. 1 Maintenance, SDS) was available *ad libitum* for all animals. During the experimental phase, time-restricted feeding was mediated by an automated FeedTime system (TSE Systems). Water was available *ad libitum* on all days for all animals. The animal handling in this study was performed in compliance with national legislation, including the 1997 Dutch Act on Animal Experimentation, and all experiments were approved by the by the National Committee for Animal experiments (CCD) and the Animal Ethics Committee of Leiden University.

### Experimental set-up

Mice were pseudo-randomly assigned to one of the five experimental groups (n = 30 per group, n = 6 per cage). A few animals were placed in a different group to ensure equal starting positions with respect to bodyweight. In a subset of mice (n = 5 per group, one animal per cage), a radio transmitter (Physio Tel, TA11 TA-F10; Data Sciences, St. Paul, MN) was implanted in the peritoneal cavity to record locomotor activity and core body temperature in 10 minute bins. After one week of recovery, each mouse was re-introduced to its previous cage.

**Figure 1:**
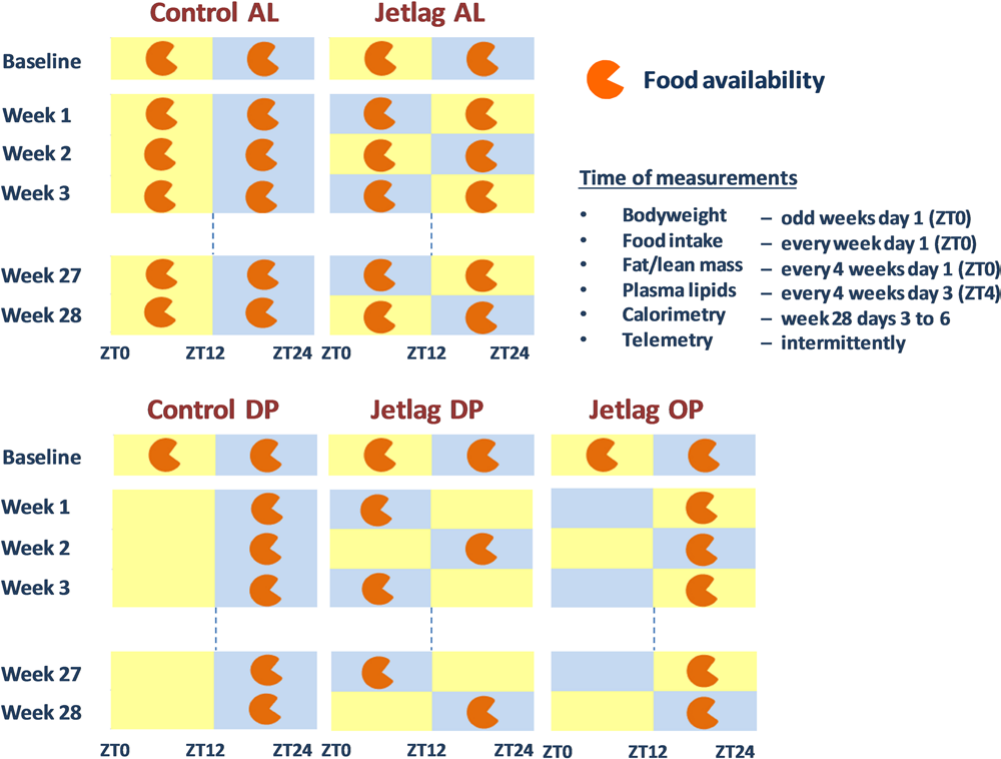
Experimental design. Effect of time-restricted feeding in animals exposed to chronic circadian rhythm disturbance (by weekly alternating light-dark cycles). Control AL = control group with normal light-dark cycle and food available *ad libitum*; Control DP = control group with normal light-dark cycle and food available during the dark phase; Jetlag AL = weekly alternating light-dark cycles (jetlag) and food available *ad libitum;* Jetlag DP = weekly alternating light-dark cycles (jetlag) and food available during the dark phase; Jetlag OP = weekly alternating light-dark cycles (jetlag) and food available during the original dark phase, i.e. in the dark phase (even weeks) or light phase (odd weeks). ZT = Zeitgeber time. ZT0 = lights on, ZT12 = lights off in control groups. Yellow background indicates lights on and blue background indicates lights off.

At 10 weeks of age, for the duration of 28 weeks, the following conditions were applied: one group of mice was exposed to normal light-dark conditions (12:12) and either fed *ad libitum* (AL) (group 1, control AL) or food availability was restricted to the dark phase (DP) (group 2, control DP). Weekly alternating light- dark cycles with a 12 h shift (jetlag) were combined with *ad libitum* feeding (group 3, jetlag AL), dark phase feeding (group 4, jetlag DP) or food available only during the original dark phase (OP) (group 5, jetlag OP), meaning that in this last group there is no shift in the timing of food availability. Thus, food is available during the dark phase in even (original) weeks and during the light phase in odd (shifted) weeks. For visual representation of the study design see Fig. 1.

### Food intake, bodyweight, estimated energy expenditure and body composition measurements

Mice were weighed in odd weeks on the first day after the shift at ZT0, when prior food exposure was the same in all groups, to determine bodyweight gain. Food was weighed weekly on the first day after the shift at ZT0, and food intake is expressed as average weekly food intake per cage per mouse, as animals were group-housed with six animals per cage. Fat and lean mass were determined with an EchoMRI-100 (Echo Medical Systems, Houston, TX) every 4 weeks, on the first day after the shift at ZT0 starting at baseline. Energy expenditure (kcal/month) was estimated by subtracting the monthly difference in fat and lean mass (kcal) from the monthly food consumption (kcal).

### Telemetric body temperature measurements

**Figure 2:**
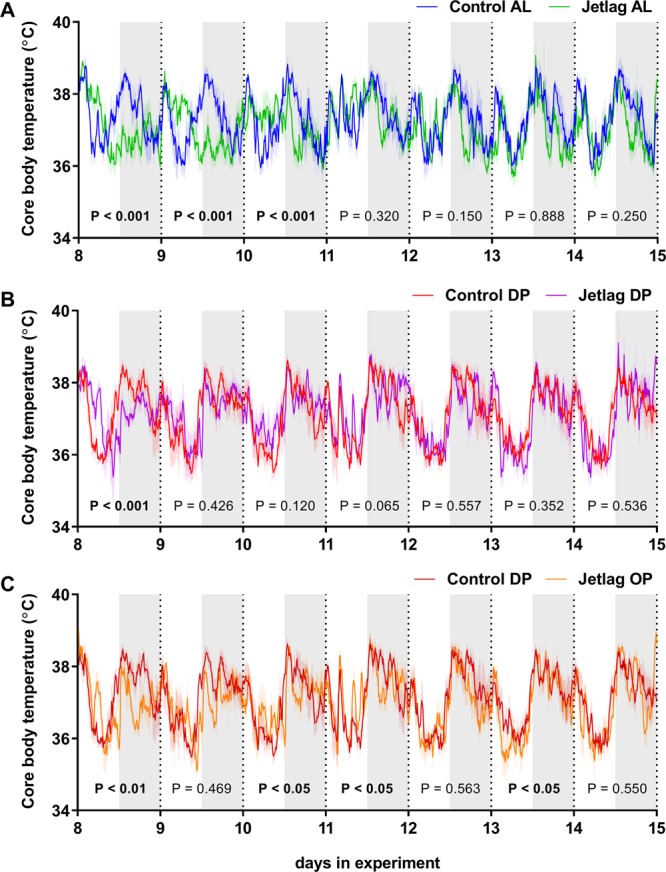
Daily core body temperature rhythms during week 2 of the experiment of the three jetlag groups compared their respective control groups **(A**–**C)**. Data are presented as means ± SEM (as indicated by lighter colored vertical lines), and grey shading indicates the dark phase. P-values represent the comparison of the within-group versus the between-group correlation (as indicated in Methods), and bold p-values indicate a significantly different temperature rhythm in the jetlag group as compared to the respective control group. Control AL = normal light-dark cycle and food available *ad libitum*; Jetlag AL = Jetlag and food available *ad libitum;* Control DP = normal light-dark cycle and food available during the dark phase; Jetlag DP = Jetlag and food available during the dark phase; Jetlag OP = Jetlag and food available alternatingly during the dark or light phase.

Body temperature was recorded using the radio transmitters for 5 days at baseline and during 3 weeks starting from the first shift. Subsequently, data was collected intermittently throughout the experiment. To investigate if time-restricted feeding can aid in adaptation to circadian rhythm disturbances, fluctuations in core body temperature were utilized as a proxy for circadian behavior. Daily rhythm of jetlag animals was compared to control animals, instead of to their own baseline, as an effect of ageing on rhythm strength was observed. This can be discerned from a decreased amplitude in core body rhythm in week 24 (Supplemental Fig. 1) as compared to week 2 (Fig. 2). Therefore, jetlag animals were compared with control animals within the same week, to prevent a confounding effect of study duration. This comparison was performed by firstly calculating the variation within the control group as references values. Thereto, each mouse within a control group was compared to all of the other mice within the same group. Core body temperature values (10 min bins) over a day were plotted against each other and Pearson’s correlation coefficients were calculated (within-group comparison). Then, core body temperatures of the jetlag mice were plotted against the values for each mouse of the respective control group, and again correlation coefficients were calculated. This resulted in one coefficient per mouse per day (between-group comparison). Finally, between-group coefficients were compared to within-group coefficients to evaluate on which day after the shift adaptation occurred in the jetlag mice. In this analysis, a ‘day’ was defined as ZT0-ZT24. Data points were only included if a reliable temperature measurement (core body temperature between 33 °C and 43 °C) was available.

### Telemetric activity measurements

Along with the body temperature recordings, ambulatory locomotor activity in the X and Y axis was collected throughout the experiment via radio transmitters. This telemetric activity data was used to predict behavioral quiescence patterns as described previously^19^. For a detailed description of this prediction method please see Juda *et al*.^20^. Briefly, telemetric activity data were collected in 10 min bins, and behavioral quiescence episodes were automatically detected in a two-step process. A trend of time series (centered moving 24 h averages) for an individual subject was calculated, and periods equal or below the within-subject threshold of 15% of that trend were considered behavioral quiescent periods. To prevent artefactual inclusions of short periods of inactivity as behavioral quiescence, we excluded sleep periods of less than three consecutive 10 min bins. In this analysis, a ‘day’ was defined as 0.00–24.00 clock time, or ZT14-ZT14. In addition, a correlation procedure was used to ignore short interruptions of behavioral quiescence periods that likely represent pronounced movements during sleep. In this procedure, the binary behavioral quiescence episodes determined as described above were correlated with a series of binary masks containing an increasing number of 1 s (behavioral quiescent bins). This mask was started at the onset of a behavioral quiescent period and filled up with 0 s (active bins) for the rest of the behavioral quiescent period. The mask that gave the highest correlation was defined as a continuous behavioral quiescent period. For more explanation on this method and examples of the calculation please see Juda *et al*.^20^.

Additionally, telemetric activity data was used to generate representative actograms using the ActogramJ software package for ImageJ^21^. F-periodograms were made using activity data of whole experimental weeks to evaluate rhythm strength, as defined by the amplitude in the periodogram (q)^22,23^. The nocturnality index (activity in the dark phase versus the active phase) was calculated to evaluate adaptation of activity rhythms per day. In these analyses, a ‘day’ was defined as ZT0-ZT24^24^.

### Indirect calorimetry

During week 28, a subset of the mice (n = 6/group) was housed individually in metabolic cages (LabMaster System, TSE Systems, Bad Homburg, Germany) for indirect calorimetry measurements. Oxygen consumption (VO_2_), carbon dioxide production (VCO_2_), food intake and ambulatory activity (beam breaks) were recorded in 20 min bins. The respiratory quotient (RQ) and energy expenditure (EE) were calculated from the VO_2_ and VCO_2_. For day 3–6 after the jetlag shift, we correlated 20 min binned group averages of the jetlagged groups to their respective control groups, the control AL group or control DP group, using Pearson’s correlation analysis.

### Plasma lipid levels

Plasma lipid levels were determined at baseline, and 4, 12 and 20 weeks after start of the experiment. Blood sampling always occurred at ZT4 on the third day after the jetlag shift and in weeks when light-dark cycles were aligned. Blood samples were collected from the tail vein of fasted ( ≥ 4 h) mice, and levels of free fatty acids (FFA), total cholesterol (TC) and triglycerides (TGs) were measured using enzymatic kits (Wako Diagnostics for FFA and Roche Diagnostics for TC/TG).

### Statistical analyses

All data are expressed as means ± standard error of the mean (SEM) and were visualized and statistically analyzed using GraphPad Prism software version 6.04 for Windows (GraphPad Software, San Diego California USA, www.graphpad.com) or R software package (R Foundation). The ChronoSapiens software was used to predict behavioral quiescence patterns^25^. One cage with 6 mice was excluded from the final analysis due to technical problems with the automatic food access roof top. Four mice were not able to complete the entire experimental procedure, due to illness, and were excluded from the final analysis (control DP: n = 1, jetlag DP: n = 1; jetlag OP: n = 2). For one cage, food intake could not be reliably determined on all time-points due to one of these ill animals. The 5 remaining mice from this cage were excluded from the analysis on cumulative food intake and estimated energy expenditure. Differences between groups in Pearson’s correlation coefficients for core body temperature were analyzed using two-sample t-tests. A p-value above 0.05 was used to define adaptation of an experimental group to its respective control group. Differences in bodyweight gain, fat mass, lean mass, estimated energy expenditure and blood lipid levels were analyzed with two-way repeated measures analysis of variance (ANOVA) followed by Sidak’s post hoc test where appropriate, to compare group differences within certain time-points. For the bodyweight gain analysis, the 0 point was not included. Differences in cumulative food intake and cumulative predicted sleep duration were analyzed with one-way ANOVA followed by Sidak’s post hoc test where appropriate. For parameters analyzed with repeated-measures ANOVA, a correction for missing values was performed using SPSS software (IBM SPSS Statistics, version 24, www. IBM.com) in which the average of two adjacent data points was taken. If these data points were not available, the animal was excluded from the analysis. Results of all statistical analyses are presented in Supplemental Table 1.

## Results

### Dark phase feeding promotes adaptation to weekly alternating light-dark cycles

To investigate if time-restricted feeding can aid in adaptation to shifts in light-dark cycle, we utilized the rhythm in core body temperature as a proxy for circadian behavior (Fig. 2). Body temperature rhythms between jetlag groups and control groups were compared, and adaptation was defined as absence of a significant difference between these two rhythms within 24 h periods (for more information on this analysis, see Methods). During week 2, in which the light-dark cycle was aligned for both jetlag and control groups, adaptation to the new light-dark cycle occurred at day 4 after the shift in the jetlag AL group (Fig. 2A). Interestingly, adaptation to the new light-dark schedule occurred around day 2 after the shift in the jetlag DP group (Fig. 2B), which is faster compared to the jetlag AL group. This indicates that restricting food access to the dark phase aids in adaptation to a new light-dark cycle. During odd weeks, when the light-dark cycle was opposite to that of controls while food availability was at the same clock time, no adaptation occurred in the jetlag OP group (Supplemental Fig. 1A). During even weeks, when light-dark cycles and food intake were aligned with the control group, adaptation occurred at a speed comparable to the jetlag DP group, although it remained more variable throughout the week (Fig. 2C). The difference in adaptation between *ad libitum* fed and dark phase fed jetlag animals persisted throughout the study, as shown by similar adaptation in week 24 as compared to week 2 (Supplemental Fig. 1B). Supplemental Table 2 contains p-values of comparisons for all weeks in which body temperature data was available.

**Figure 3:**
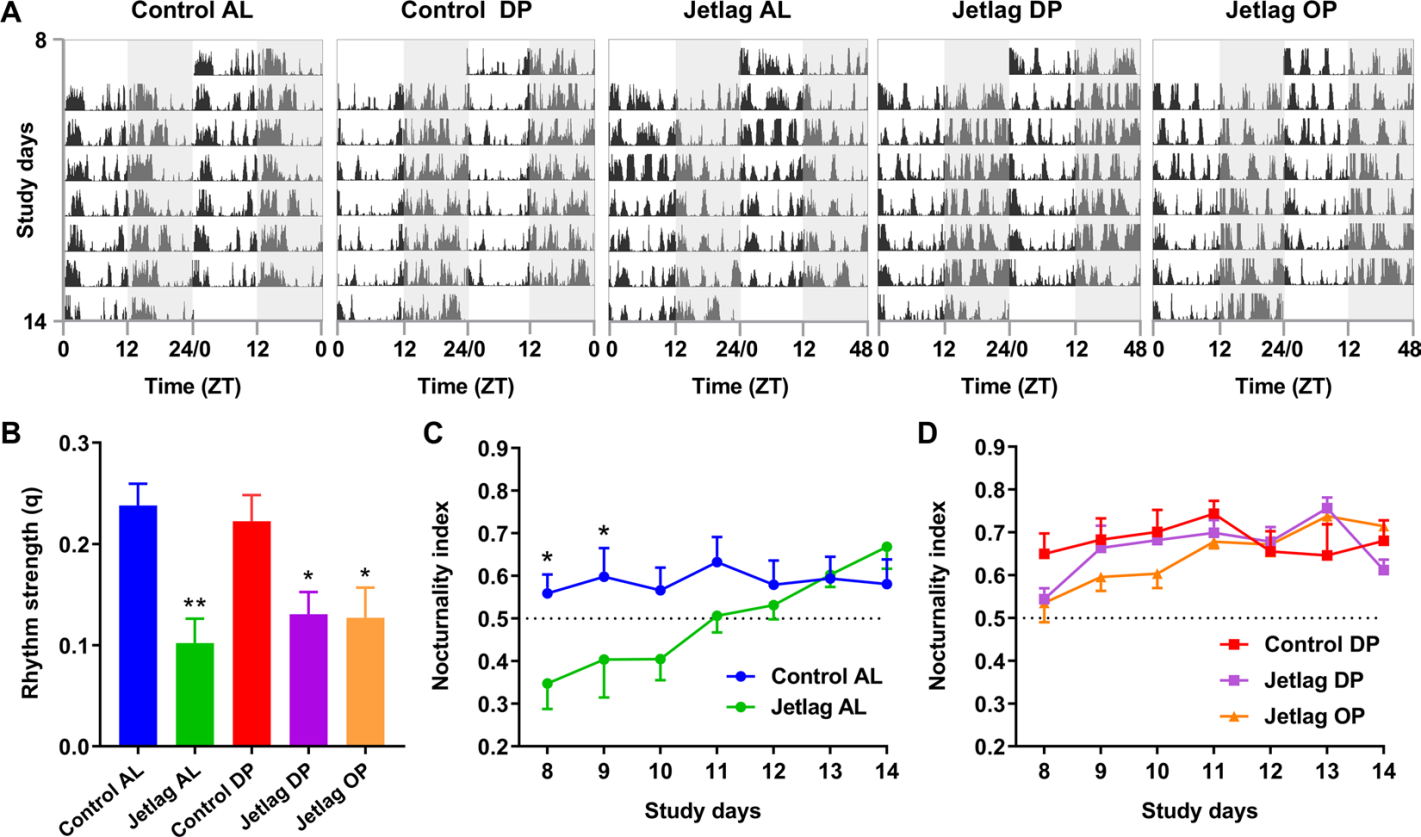
Representative double-plotted actograms of week 2 of the study are shown, in which grey shading indicates the dark period **(A)**. F-periodogram analysis was performed to calculate rhythm strength (q) of activity over the whole week **(B)**, and the nocturnality index (activity in the dark versus light phase) was calculated to evaluate rhythm strength per day **(C**,**D)**. Control AL = normal light-dark cycle and food available *ad libitum*; Jetlag AL = Jetlag and food available *ad libitum;* Control DP = normal light-dark cycle and food available during the dark phase; Jetlag DP = Jetlag and food available during the dark phase; Jetlag OP = Jetlag and food available alternatingly during the dark or light phase. *p < 0.05, **p < 0.01 vs. control AL. Statistical differences between other groups are not indicated, please see Supplemental Table 1.

Next, we aimed to substantiate these results by evaluating rhythm in ambulatory locomotor activity as another proxy for circadian behavior, as illustrated by actograms in Fig. 3A. As expected, the rhythm strength of activity in week 2 was decreased in all jetlag mice as compared to controls (Fig. 3B). However, no significant improvement was observed by time-restricted feeding when evaluating the compiled data of week 2. Calculating the nocturnality index (activity in dark versus light phase) as a measure of rhythm strength per day did reveal differences between *ad libitum* and time-restricted feeding. While jetlag AL mice require around 4 days to adapt their nocturnality index to that of control animals (Fig. 3C), dark phase fed mice show adaptation almost instantly after the shift in light-dark cycle (Fig. 3D). This difference in adaptation of activity rhythms persisted throughout the study (Supplemental Fig. 2), similar to the observed adaptation of core body temperature.

To assess adaptation of metabolic parameters to alternating light-dark cycles, a subset of animals (n = 6 per group) was individually placed in metabolic cages for indirect calorimetric phenotyping during week 28 of the experiment (Supplemental Figs 3 and 4), when light-dark schedules were aligned between groups. Oxygen consumption and the respiratory quotient correlated more strongly in the jetlag DP and control DP groups as compared to the jetlag AL and control AL group from day 3–6 (Table 1). This suggests that adaptation of daily rhythms in oxygen consumption and the respiratory quotient occurred faster when food was restricted to the dark phase, as was observed for daily patterns of core body temperature and ambulatory locomotor activity. Adaptation in ambulatory locomotor activity as assessed by beam breaks in the metabolic cage system also appeared stronger in the jetlag DP group as compared to the jetlag AL group (Table 1), although this difference was less pronounced. However, since calorimetry data was not collected on day 1 and 2 after the shifts, conclusion on accelerated adaptation must be drawn with caution. We further looked into the ambulatory locomotor activity and calculated the cumulative activity during the period of data collection (day 3–6 after shift). During these days, no significant differences in total cumulative activity were observed between groups (p = 0.7019; Supplemental Fig. 4; Supplemental Table 1).

**Table 1:** Correlation coefficient of daily patterns (20 min bins) in oxygen consumption (VO_2_ (ml/h)), energy expenditure (kcal/h), the respiratory quotient and ambulatory locomotor activity (beam breaks) during week 28.

Days after shift	Oxygen consumption			Energy expenditure			Respiratory quotient			Locomotor activity		
	jetlag AL vs. control AL	jetlag DP vs. control DP	Jetlag OP vs. control DP	jetlag AL vs. control AL	jetlag DP vs. control DP	jetlag OP vs. control DP	jetlag AL vs. control AL	jetlag DP vs. control DP	jetlag OP vs. control DP	jetlag AL vs. control AL	jetlag DP vs. control DP	jetlag OP vs. control DP
3	0.102	0.596	0.429	0.111	0.598	0.466	0.161	0.981	0.99	0.175	0.379	0.270
4	0.565	0.618	0.622	0.600	0.630	0.635	0.551	0.973	0.978	0.308	0.441	0.667
5	0.474	0.671	0.733	0.562	0.709	0.757	0.697	0.940	0.976	0.458	0.474	0.575
6	0.604	0.598	0.81	0.680	0.674	0.841	0.924	0.976	0.982	0.454	0.611	0.617

Jetlagged groups are compared to their respective control groups: jetlag AL vs. control AL, jetlag DP vs. control DP and jetlag OP vs. control DP. For the jetlag OP group food is available during the dark phase during this week.

In summary, daily patterns of core body temperature and ambulatory locomotor activity throughout the experiment and daily patterns of oxygen consumption and the respiratory quotient at the end of the experiment (after 28 weeks) indicate that aligning food intake to the dark phase during weekly alternating light-dark cycles promotes adaptation to the new light-dark schedule.

### Weekly alternating light-dark cycles combined with food restriction to the dark phase decreases predicted behavioral quiescence

**Figure 4:**
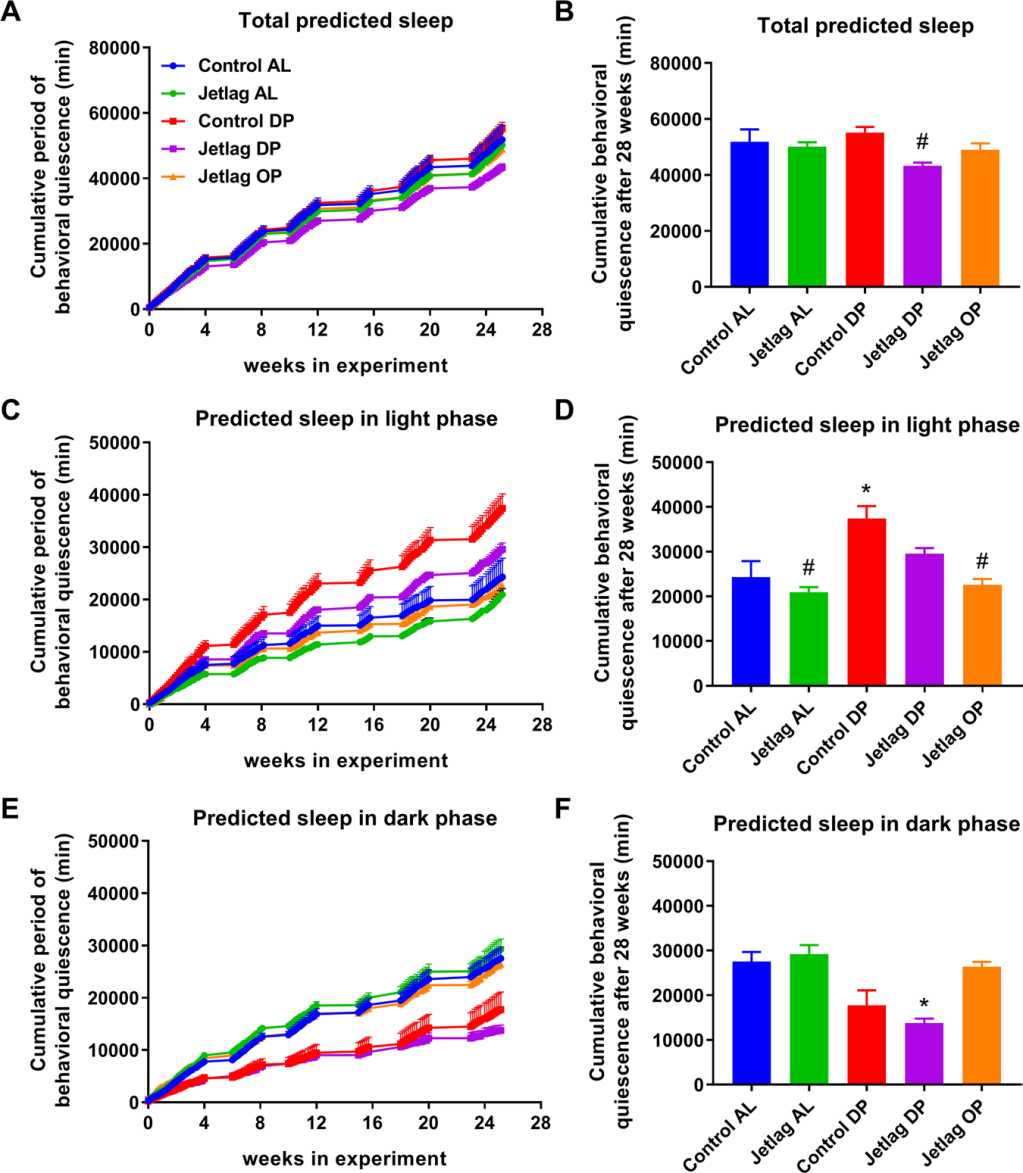
Cumulative predicted sleep during both the light and dark phases **(A**,**B)**, only the light phases **(C**,**D)** and only the dark phases **(E**,**F)**. Panels (B,D,F) show total cumulative predicted sleep after 28 weeks with 92 data collection days (due to intermittent data collection; 47 days during even weeks and 45 days during odd weeks). Control AL = normal light-dark cycle and food available *ad libitum*; Jetlag AL = Jetlag and food available *ad libitum;* Control DP = normal light-dark cycle and food available during the dark phase; Jetlag DP = Jetlag and food available during the dark phase; Jetlag OP = Jetlag and food available alternatingly during the dark or light phase. *p < 0.05 vs. control AL. ^#^p < 0.05 vs. control DP. Statistical differences between other groups are not indicated, please see Supplemental Table 1.

Next, we calculated cumulative levels of behavioral quiescence using telemetric activity data, as a measure of predicted sleep. Behavioral quiescence was determined over 27 weeks during the light phases, dark phases and both phases combined (Fig. 4). Of note, there were no clear differences in predicted sleep between different weeks of the study, also when comparing even versus odd weeks (Supplemental Fig. 5). Total cumulative behavioral quiescence was decreased in the jetlag DP group compared to its control group (i.e. control DP, Fig. 4B; p = 0.0358). This indicates that when weekly alternating light-dark schedules are combined with time-restricted feeding to the dark phase, total predicted sleep is decreased. This effect is not observed when only one of these experimental conditions is present (e.g. control AL vs. jetlag AL or control AL vs. control DP).

As may be expected, significant differences were also observed in the timing of behavioral quiescence (Fig. 4C–F; Supplemental Table 1). Behavioral quiescence during the light phase was increased in the control DP group compared to the jetlag AL group (p = 0.0055). In line with this finding, behavioral quiescence is decreased during the dark phase in the jetlag DP group (Fig. 4D,F; p = 0.0061 vs. control AL, p = 0.0011 vs. jetlag AL; Supplemental Table 1). Thus, food restriction to the dark phase of animals in the control DP and jetlag DP groups seemed to change their timing of predicted sleep. However, for the jetlag DP group this was not sufficient to get total predicted sleep duration completely compensated.

### Weekly alternating light-dark cycles and time-restricted feeding reduce estimated energy expenditure

**Figure 5:**
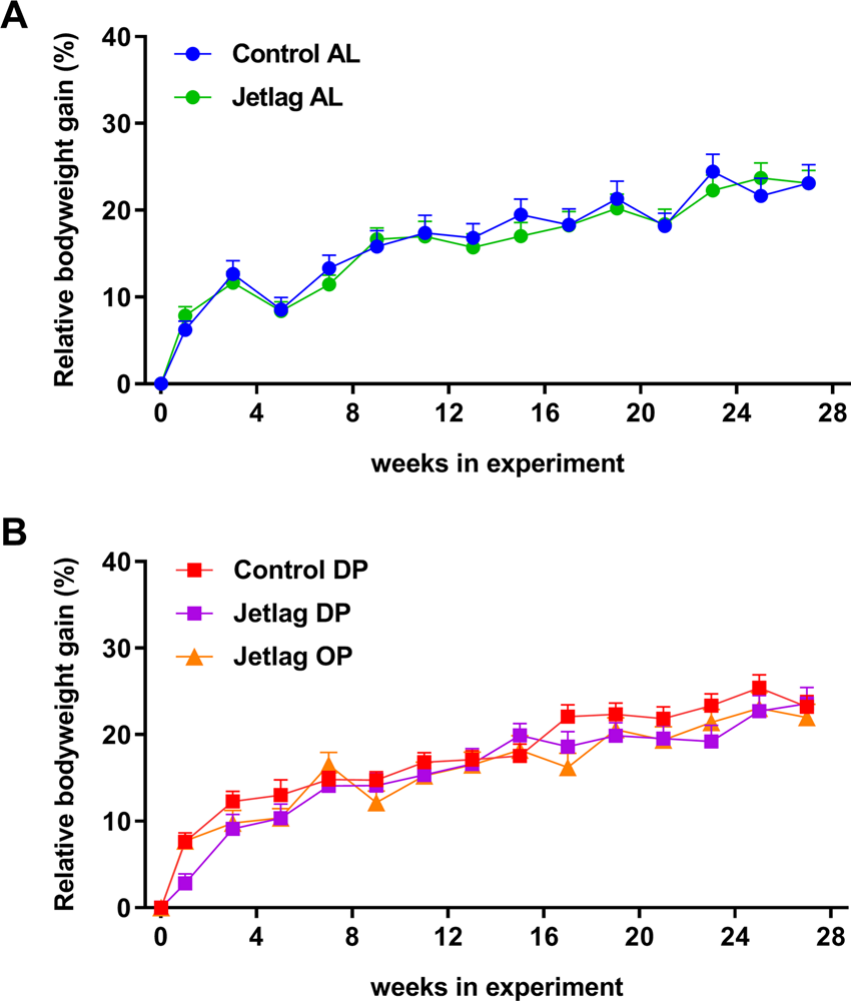
Relative bodyweight gain in *ad libitum* fed animals **(A)** and food restricted animals **(B)**, expressed as percentage increase in bodyweight relative to baseline at time points were prior food availability was equal in all groups. Control AL = normal light-dark cycle and food available *ad libitum*; Jetlag AL = Jetlag and food available *ad libitum*. Control DP = normal light-dark cycle and food available during the dark phase; Jetlag DP = Jetlag and food available during the dark phase; Jetlag OP = Jetlag and food available alternatingly during the dark or light phase.

**Figure 6:**
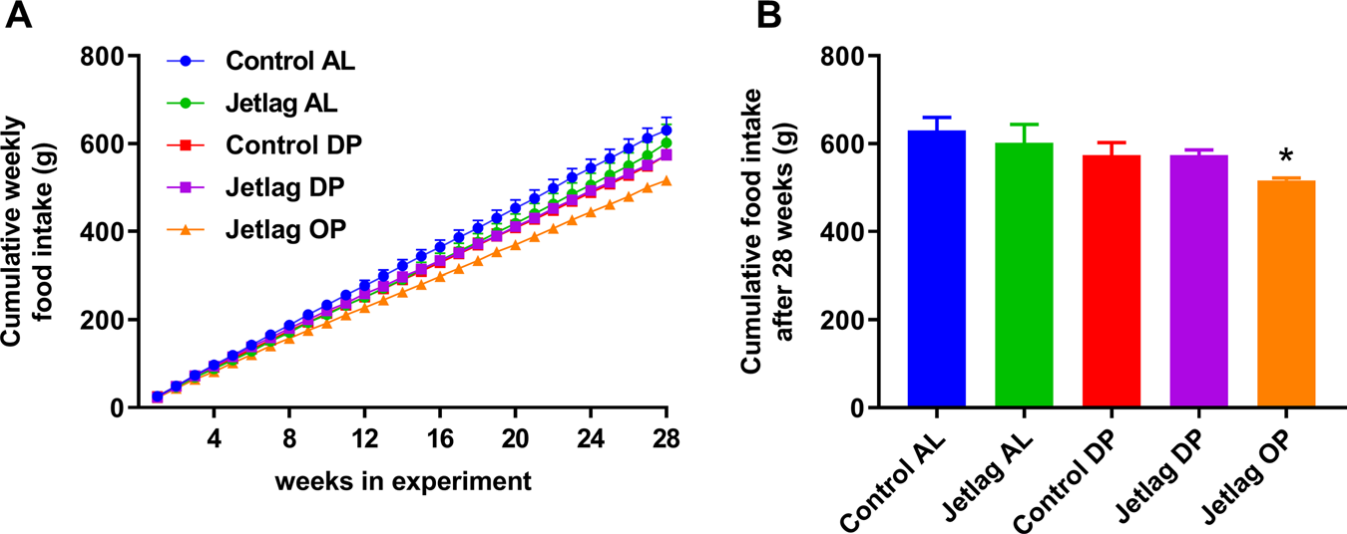
Cumulative weekly food intake **(A)** and total cumulative food intake after 28 weeks **(B)** in average grams per group. Weekly food intake was measured per cage per mouse. Control AL = normal light-dark cycle and food available *ad libitum*; Jetlag AL = Jetlag and food available *ad libitum;* Control DP = normal light-dark cycle and food available during the dark phase; Jetlag DP = Jetlag and food available during the dark phase; Jetlag OP = Jetlag and food available alternatingly during the dark or light phase. * indicates p < 0.05 vs. control AL group.

**Figure 7:**
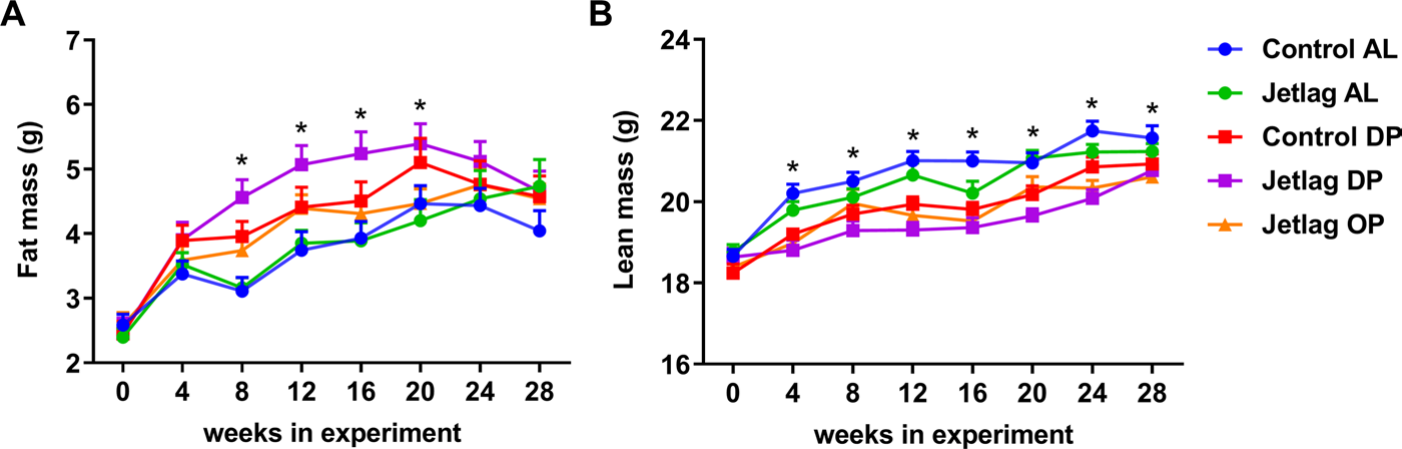
Body composition measurements of fat mass (**A**) and lean mass (**B**). Control AL = normal light-dark cycle and food available *ad libitum*; Jetlag AL = Jetlag and food available *ad libitum;* Control DP = normal light-dark cycle and food available during the dark phase; Jetlag DP = Jetlag and food available during the dark phase; Jetlag OP = Jetlag and food available alternatingly during the dark or light phase. * indicates statistically significant difference between groups (p < 0.05), please see text and Supplemental Table 1 for further details.

Next, we investigated whether the experimental groups differed in bodyweight, body composition, cumulative food intake or estimated energy expenditure. There was no significant difference between the groups in relative bodyweight gain at all time points where food exposure prior to measurement of bodyweight was equal (Fig. 5; Supplemental Table 1). Total cumulative food intake after 27 weeks did differ between experimental groups (Fig. 6; p = 0.0214). Post hoc testing revealed a significant difference between the jetlag OP and control AL groups (p = 0.0137, Supplemental Table 1), suggesting a combined effect of time-restricted feeding and jetlag. Restricting food to the dark phase during jetlag exposure (jetlag DP) appears to increase fat mass, compared to *ad libitum* fed animals exposed to normal light-dark cycles (control AL) or to jetlag (jetlag AL) (Fig. 7A; Supplemental Table 1). In addition, time-restricted feeding had an effect on lean mass (Fig. 7B; p < 0.001; Supplemental Table 1), since lean mass was reduced in all timed food restricted groups (control DP, jetlag DP, and jetlag OP) relative to the *ad libitum* groups (control AL and jetlag AL).

**Figure 8:**
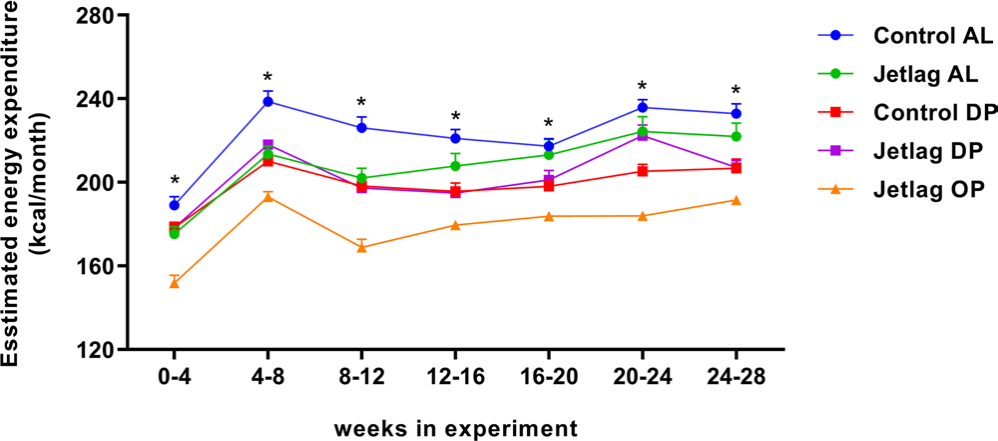
Estimated energy expenditure (kcal/month) calculated as monthly food intake (kcal) minus monthly difference in fat and lean mass (kcal). Control AL = normal light-dark cycle and food available *ad libitum*; Jetlag AL = Jetlag and food available *ad libitum;* Control DP = normal light-dark cycle and food available during the dark phase; Jetlag DP = Jetlag and food available during the dark phase; Jetlag OP = Jetlag and food available alternatingly during the dark or light phase. * indicates statistically significant difference between groups (p < 0.05), please see text and Supplemental Table 1 for further details.

In the jetlag OP group, food intake was decreased while bodyweight remained unchanged, suggesting a decreased energy expenditure. To validate this, energy expenditure (in kcal/month) was estimated by subtracting the monthly difference in fat and lean mass from the monthly food consumption. Indeed, estimated energy expenditure for the jetlag OP group was decreased compared to all other experimental groups (Fig. 8; Supplemental Table 1). Furthermore, all other experimental groups showed a reduced energy expenditure compared to the control AL group, although less profound. Both weekly alternating light-dark cycles and restricted food access during the dark phase reduced energy expenditure (Fig. 8; Supplemental Table 1).

Taken together, these results indicate only modest effects on body composition and food intake by time-restricted feeding alone or in combination with weekly alternating light-dark cycles. However, combining the two measurements in estimated energy expenditure does point to metabolic alterations induced by both jetlag and time-restricted feeding, being most pronounced in the jetlag OP group.

### Time-restricted feeding, but not weekly alternating light-dark schedules, affect plasma lipid levels

**Figure 9:**
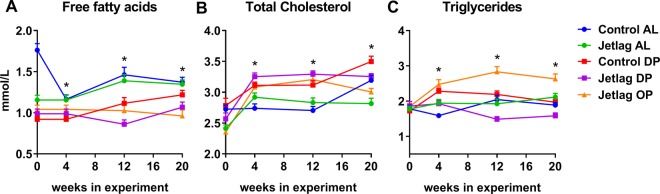
Plasma levels of free fatty acids (**A**), total cholesterol (**B**) and triglycerides (**C**). Plasma lipids levels were measured at baseline (week 0), after 4 weeks, after 12 weeks and after 20 weeks. Control AL = normal light-dark cycle and food available *ad libitum*; Jetlag AL = Jetlag and food available *ad libitum*; Control DP = normal light-dark cycle and food available during the dark phase; Jetlag DP = Jetlag and food available during the dark phase; Jetlag OP = Jetlag and food available alternatingly during the dark or light phase. * indicates statistically significant difference between groups (p < 0.05), please see text and Supplemental Table 1 for further details.

To further study metabolic phenotypes, plasma levels of FFAs, TC, and TGs were determined at ZT4 in fasted mice at day 3 of every 4^th^ week. Levels of FFAs were decreased in all time-restricted feeding groups (control DP, jetlag DP, jetlag OP) compared to the *ad libitum* groups (Fig. 9A, p < 0.0001; Supplemental Table 1). This effect was most apparent after 12 weeks of exposure, but somewhat smaller after 20 weeks. TC levels were increased in all time-restricted feeding groups compared to the *ad libitum* groups (Fig. 9B, p < 0.001; Supplemental Table 1). Similar to FFAs, this effect was most apparent after 12 weeks of exposure and was less profound (control DP vs. control AL) or absent after 20 weeks (jetlag DP and jetlag OP vs. control AL). Patterns of plasma TG levels were different from those of FFA and TC. No effect of time-restricted feeding alone was observed, but plasma levels of TGs were decreased in the jetlag DP group and increased in the jetlag OP group (Fig. 9C; p < 0.0001; Supplemental Table 1). This again suggests a combined effect of jetlag and food restriction.

Taken together, these results indicate that time-restricted feeding decreases plasma FFA levels and increases plasma TC levels. No effects of weekly alternating light-dark cycles alone were observed on any of the measured lipids. Interestingly, TG levels were affected by the combination of time-restricted feeding and jetlag. Although the light-dark cycle and timing of food availability was the same for all groups at the moment of blood sampling, alternating access to food during the light or dark phase combined with jetlag (jetlag OP) resulted in increased TG levels, whereas access to food during the dark phases combined with jetlag (jetlag DP) resulted in decreased TG levels.

## Discussion

### Effects on adaptation to weekly alternating light-dark schedules

In this study, we investigated the effect of time-restricted feeding for 12 h on adaptation to weekly alternating light-dark cycles (12 h shift). We observed that time-restricted feeding has an effect on adaptation to a new light-dark schedule. Restricting food access to the dark phase enhanced adaptation of core body temperature and ambulatory locomotor activity rhythms by 2–3 days as compared to *ad libitum* feeding. In contrast, when food availability was not constantly aligned to the dark cycle (jetlag OP group) adaptation was highly variable. It appeared that during weeks when food intake was restricted to the light phase, core body temperature patterns remained more aligned with the food availability schedule rather than the light-dark schedule. However, this was not observed in all weeks throughout the experiment, making it difficult to draw firm conclusions.

For the jetlag DP group, similar effects on adaptation were observed for metabolic parameters, such as oxygen consumption and the respiratory quotient at the end of the experiment (after 28 weeks). However, as metabolic data for day 1 and 2 after the shift was not available, no firm conclusion can be drawn on the speed of adaptation for these parameters. Nevertheless, collectively these results indicate that aligning food intake to the new dark (active) phase expedites adaptation to a 12 h shift in the light-dark schedule and that this effect remains present over months. These findings are in line with previous studies showing enhanced adaptation to 6 h advancing light-dark schedules with dark phase restricted feeding^26–28^. In these studies, food availability was restricted to 2 h during the dark phase. We demonstrate that food restriction to the entire dark phase also enhances adaptation, even with a larger advance in light-dark schedules (12 h). Additionally, in contrast to the previous studies where animals were investigated for 5 days, we maintained animals on the imposed schedules for 28 weeks. Our results suggest that time-restricted feeding has an ongoing effect on adaptation, independent of the duration of rhythm disturbance. It is important to note that there are differences in the method of analyzing adaptation between the described studies as well as differences in study design, which makes direct comparisons between studies difficult. Nevertheless, all studies support the notion that timing of food availability can accelerate adaption to changed light-dark schedules of different parameters such as core body temperature and locomotor activity.

When mice were fed at a fixed 12 h but the light-dark schedule was reversed (jetlag OP group in this study) animals did not adapt their core body temperature rhythms to this new light-dark schedule. Interestingly, they did not completely keep their original 24 h rhythm in core body temperature either. Previously, it has been reported that restricting food availability to the light phase, affects the shape, amplitude and phase of core body temperature rhythms of mice^29^. Main changes observed are the occurrence of a biphasic pattern with peaks around lights on and lights off and a large dip in body temperature during the dark phase. In the current study, we observed similar changes in patterns of core body temperature during days with restricted food availability during the light phase, however, these effects were much less profound (Supplemental Fig. 1). This could be related to the more cathemeral nature of FVB animals, with the control group (control AL) also showing a somewhat biphasic rhythm of core body temperature (Supplemental Fig. 1) and a relative large proportion of sleep during the dark phase (53% of cumulative predicted sleep duration over 92 days in the control AL group as discussed below). It has been described before that FVB mice are less nocturnal as compared to C57BL/6 J mice, as indicated by a relatively small difference in the amount of food intake and physical activity in the day versus night^30^. Circadian activity patterns in rodents are driven by evolution, and fine-tuned by interactions with the environment (*e*.*g*. food availability, predator/prey interactions), which has resulted in differences between rodent strains and species^31^. For example, the Algerian mouse is mainly nocturnal, but shows biphasic activity patterns in spring and autumn^32^, similar to the activity patterns of FVB mice in our study. How these differences in activity patterns (*i*.*e*. diurnal, nocturnal, or multiphasic) affect the speed of adaptation remains largely unknown, and would be an interesting subject for further investigation.

### Effects on predicted sleep

To our knowledge, this is the first study investigating the effect of time-restricted feeding on predicted sleep patterns by evaluating periods of behavioral quiescence. We observed that, when food is restricted to the dark phase, the duration of predicted sleep is reduced during the dark phase and increased during the light phase, resulting in a net equal amount of predicted sleep. When timing of food restriction during the dark phase is combined with weekly alternating light-dark schedules, it appears that this compensation of predicted sleep during the light phase is insufficient, resulting in a net decreased amount of predicted sleep. It is known that FVB animals sleep a relatively large portion of their total sleep during the dark phase, as was previously determined using EEG measurements in FVB male mice^33^. The differences in predicted sleep timing observed in the control DP and jetlag DP groups when compared to the *ad libitum* fed groups are therefore likely related to this cathemeral nature of FVB animals, where time-restricted feeding to the dark phase interferes with their normal sleeping pattern. This cumulative effect on predicted sleep timing and total predicted sleep duration is not present in the jetlag OP group, in which food access alternates weekly between the dark and light phase. This indicates that biweekly time-restricted feeding to the dark phase is not sufficient to disturb cumulative predicted sleep duration and timing when both types of weeks are taken into account.

Previously, we observed that weekly alternating light-dark cycles cause increased predicted sleep duration in *p53*^*R270H/*+^ *WAPCre* female FVB mice^19^. In the current study, this effect was not observed. This might indicate that the genetic background might play a role in the regulation of sleep or circadian rhythms. The link between circadian clock regulation of the cell cycle and tumor progression via p53 is often reported^34^, however, literature regarding a possible role for p53 in regulating physiological or behavioral diurnal rhythms is scarce. As a possible link, p53 knock-out mice show less robust diurnal rhythms in drinking pattern^35^. However, when core body temperature rhythms of the control animals in our study (control AL) were compared to the control animals of the previous study (*p53*^*R270H/*+^ *WAPCre* mice) under baseline conditions (4 days), curves were quite comparable (correlation coefficient > 0.69). This indicates no obvious differences in one parameter of circadian rhythms between the two types of mice, but since data was collected in two separate experiments this conclusion must be drawn with caution.

### Effects on metabolic parameters

Previously, we observed that weekly alternating light-dark cycles increases bodyweight in *p53*^*R270H/*+^ *WAPCre* female FVB mice^19^. In the current study we observed no changes in most metabolic parameters (bodyweight, food intake, body composition and plasma lipid levels) due to the weekly alternating light-dark schedules when food was available *ad libitum*. This discrepancy could be explained by *p53*^*R270H/+*^ *WAPCre* female FVB mice being more prone to metabolic changes, as loss of function of p53 has been related to altered lipid metabolism and obesity^36,37^. However, the lack of bodyweight gain in the current study is also not consistent with other studies demonstrating an effect of chronic rhythm disturbance (i.e. by phase advances or 12 h shifts of the photoperiod) on bodyweight in various rodent models^10,38–41^. This discrepancy cannot be explained by a difference in species or diet, as some of these studies were also performed in mice on a chow diet similar to our study^38,40^. However, while these studies have used male C57BL/6 mice to study metabolic effects of rhythm disturbances, we have used female FVB mice. Therefore, differences in metabolism between gender and various mouse strains such as C57BL/6 and FVB mice could have influenced the study outcome^42,43^.

In the current study, we did observe effects of restricting food to the dark phase under a normal light-dark schedule on lean mass, energy expenditure and lipid levels. It cannot be excluded that food intake or increased light exposure prior to the measurements has influenced these data, in particular for the lipid levels. Nonetheless, body temperature rhythms indicated adaptation on day 3 after the shift in jetlag groups at the even weeks when blood was drawn (i.e. week 4, 12 and 20), suggesting that rhythms were in phase at the time of blood drawing. In the jetlag DP group, plasma levels of TG were decreased, suggesting a more favorable metabolic state. However, an increase in fat mass, a reduction in lean mass and reduced estimated energy expenditure were also observed. In the jetlag OP group, increased plasma TG levels, reduced food intake and reduced estimated energy expenditure were observed, without changes in bodyweight, indicating a somewhat less favorable metabolic state. In addition, the jetlag OP group displayed a disturbed adaption to the new-light dark schedule during the weeks that food is available during the light phase. This might indicate that impairing adaptation to a new light-dark schedule by time-restricted feeding increases metabolic risks. In this study, there appears to be no clear role for predicted sleep duration or timing in this effect, since there was no change in periods of behavioral quiescence in the jetlag OP group.

There are several previous studies in which a model for chronic circadian rhythm disturbance was combined with restricted timing of food intake^10,38,40^. In those studies, rhythm disturbance was caused by a forced period of activity during the normal resting phase or by a 6 h advance in light-dark schedules every 2 days or twice a week. In all studies, an effect of chronic rhythm disturbance on several metabolic parameters was observed, including increased bodyweight. Food restriction to the inactive light period worsened metabolic health^10^. This is in line with the less favorable metabolic state observed in the jetlag OP group in our study, which was fed in the inactive phase every other week. However, another study showed that food restriction to a fixed time (partly in the inactive period and partly in the active period) alleviates metabolic effects of circadian rhythm disturbances^40^, which is not in accordance with our results. Food restriction to the normal active period has been shown to improve metabolism when rhythm was disturbed^10,38^. These results are also not in accordance with our study, as we did not observe a positive effect on most metabolic parameters in dark phase fed animals (jetlag DP group).

These discrepancies suggest that small variations in study setup (e.g. variations in light-dark schedule, gender, genetic background, etc.) could result in different outcomes. This is also observed for humans, as some shift workers have less complaints than others, depending, for example, on the chronotype of an individual. More specifically, a recent study observed an increased risk of overweight in selectively shift workers with an evening chronotype instead of a morning chronotype^44^. In addition, various types of shift work schedules exist, and some are better tolerated than others^45,46^. Thus, although we performed a long-term study with large group sizes, our specific study conditions did not result in remarkable metabolic alterations.

In summary, this study shows that time-restricted feeding has an effect on adaptation to changing light-dark schedules, with accelerated adaptation of core body temperature and locomotor activity rhythms when food is provided during the dark phase. Time-restricted feeding alone and/or in combination with weekly alternating light-dark schedules causes very mild changes in metabolic parameters, such as lean mass, energy expenditure and lipid levels, and results in altered timing and duration of predicted sleep. The least favorable metabolic state is observed in the jetlag OP group. In this group, adaptation to the new light-dark schedule is disturbed, in particular when the light-dark cycle and food availability are misaligned. Jetlag DP mice adapted best to a new light-dark cycle, however, this was not accompanied by a significant improvement of metabolic parameters.

Thus, our results demonstrate that dietary interventions could be a promising strategy to expedite adaptation of the circadian system. However, future research in alternative animal models is needed to demonstrate whether dark phase feeding could improve metabolic health by enhancing adaptation. If so, this could be a promising strategy to influence adverse health outcomes in circadian rhythm disturbances, such as shift work or jetlag.

## Supplementary information

Supplementary Dataset 1

## Data Availability

The datasets generated during and/or analyzed during the current study are available from the corresponding author on reasonable request.
