# Supplementary material for: Time-restricted feeding improves adaptation to chronically alternating light-dark cycles
Source: Sci Rep. 2019 May 27;9:7874. doi: 10.1038/s41598-019-44398-7 (PMC6536683; doi:10.1038/s41598-019-44398-7)
Supplement: Supplementary file 1 — Supplementary Dataset 1 [file 41598_2019_44398_MOESM1_ESM.docx]

**Supplemental data to:**

**Time-restricted feeding improves adaptation to chronically alternating light-dark cycles**

Maaike Schilperoort, Rosa van den Berg, Martijn E.T. Dollé, Conny T.M. van Oostrom, Karina Wagner, Lauren L. Tambyrajah, Paul Wackers, Tom Deboer, Gerben Hulsegge, Karin I. Proper, Harry van Steeg, Till Roenneberg, Nienke R. Biermasz, Patrick C.N. Rensen, Sander Kooijman, Linda W.M. van Kerkhof

**Supplemental Tables**

**Supplemental Table 1** Values for all statistical tests described in this paper.

| **Parameter** | **ANOVA** | **Post hoc tests** | **Adjusted p-value** |
| --- | --- | --- | --- |
| Rhythm strength  (week 2) | Group F (4, 20) = 6.107, **p = 0.0022** | Control AL vs. Control DP  Control AL vs. Jetlag AL  Control AL vs. Jetlag DP  Control AL vs. Jetlag OP  Control DP vs. Jetlag AL  Control DP vs. Jetlag DP  Control DP vs. Jetlag OP  Jetlag AL vs. Jetlag DP  Jetlag AL vs. Jetlag OP  Jetlag DP vs. Jetlag OP | 0.9915  **0.0078**  **0.0451**  **0.0365**  **0.0207**  0.1081  0.0890  0.9244  0.9522  >0.9999 |
| Rhythm strength (week 24) | Group F (4, 19) = 7.921, **p = 0.0006** | Control AL vs. Control DP  Control AL vs. Jetlag AL  Control AL vs. Jetlag DP  Control AL vs. Jetlag OP  Control DP vs. Jetlag AL  Control DP vs. Jetlag DP  Control DP vs. Jetlag OP  Jetlag AL vs. Jetlag DP  Jetlag AL vs. Jetlag OP  Jetlag DP vs. Jetlag OP | 0.6536  0.1631  0.2168  **0.0092**  **0.0140**  0.0197  **0.0007**  0.9998  0.6189  0.5180 |
| Nocturnality index  (week 2) | Group F (4, 19) = 3.678, **p = 0.0222**  time F (6, 114) = 14.87, **p < 0.0001**  time x group F (24, 114) = 3.846, **p < 0.0001** | Day 8  Control AL vs. Control DP  Control AL vs. Jetlag AL  Control AL vs. Jetlag DP  Control AL vs. Jetlag OP  Control DP vs. Jetlag AL  Control DP vs. Jetlag DP  Control DP vs. Jetlag OP  Jetlag AL vs. Jetlag DP  Jetlag AL vs. Jetlag OP  Jetlag DP vs. Jetlag OP  Day 9  Control AL vs. Control DP  Control AL vs. Jetlag AL  Control AL vs. Jetlag DP  Control AL vs. Jetlag OP  Control DP vs. Jetlag AL  Control DP vs. Jetlag DP  Control DP vs. Jetlag OP  Jetlag AL vs. Jetlag DP  Jetlag AL vs. Jetlag OP  Jetlag DP vs. Jetlag OP  Day 10  Control AL vs. Control DP  Control AL vs. Jetlag AL  Control AL vs. Jetlag DP  Control AL vs. Jetlag OP  Control DP vs. Jetlag AL  Control DP vs. Jetlag DP  Control DP vs. Jetlag OP  Jetlag AL vs. Jetlag DP  Jetlag AL vs. Jetlag OP  Jetlag DP vs. Jetlag OP  Day 11  Control AL vs. Control DP  Control AL vs. Jetlag AL  Control AL vs. Jetlag DP  Control AL vs. Jetlag OP  Control DP vs. Jetlag AL  Control DP vs. Jetlag DP  Control DP vs. Jetlag OP  Jetlag AL vs. Jetlag DP  Jetlag AL vs. Jetlag OP  Jetlag DP vs. Jetlag OP  Day 12  Control AL vs. Control DP  Control AL vs. Jetlag AL  Control AL vs. Jetlag DP  Control AL vs. Jetlag OP  Control DP vs. Jetlag AL  Control DP vs. Jetlag DP  Control DP vs. Jetlag OP  Jetlag AL vs. Jetlag DP  Jetlag AL vs. Jetlag OP  Jetlag DP vs. Jetlag OP  Day 13  Control AL vs. Control DP  Control AL vs. Jetlag AL  Control AL vs. Jetlag DP  Control AL vs. Jetlag OP  Control DP vs. Jetlag AL  Control DP vs. Jetlag DP  Control DP vs. Jetlag OP  Jetlag AL vs. Jetlag DP  Jetlag AL vs. Jetlag OP  Jetlag DP vs. Jetlag OP  Day 14  Control AL vs. Control DP  Control AL vs. Jetlag AL  Control AL vs. Jetlag DP  Control AL vs. Jetlag OP  Control DP vs. Jetlag AL  Control DP vs. Jetlag DP  Control DP vs. Jetlag OP  Jetlag AL vs. Jetlag DP  Jetlag AL vs. Jetlag OP  Jetlag DP vs. Jetlag OP | Day 8  0.8135  **0.0202**  >0.9999  >0.9999  **0.0001**  0.6441  0.5284  **0.0396**  0.0589  >0.9999  Day 9  0.8650  **0.0437**  0.9719  >0.9999  **0.0006**  >0.9999  0.8440  **0.0017**  **0.0485**  0.9641  Day 10  0.2978  0.1621  0.5109  0.9997  **0.0002**  >0.9999  0.7380  **0.0006**  **0.0356**  0.9127  Day 11  0.5686  0.4805  0.9695  0.9982  **0.0057**  0.9986  0.9730  **0.0475**  0.1107  >0.9999  Day 12  0.9262  0.9985  0.7194  0.7972  0.4985  >0.9999  >0.9999  0.2654  0.3285  >0.9999  Day 13  0.9950  >0.9999  0.1073  0.2194  0.9993  0.5812  0.8013  0.2114  0.3750  >0.9999  Day 14  0.7171  0.8837  >0.9999  0.3114  >0.9999  0.9634  0.9999  0.9939  0.9989  0.6786 |
| Nocturnality index  (week 24) | Group F (4, 131) = 11.83, **p < 0.0001**  time F (6, 131) = 1.841, p = 0.0959  time x group F (24, 131) = 0.6155, p = 0.9165 | Day 163  Control AL vs. Control DP  Control AL vs. Jetlag AL  Control AL vs. Jetlag DP  Control AL vs. Jetlag OP  Control DP vs. Jetlag AL  Control DP vs. Jetlag DP  Control DP vs. Jetlag OP  Jetlag AL vs. Jetlag DP  Jetlag AL vs. Jetlag OP  Jetlag DP vs. Jetlag OP  Day 164  Control AL vs. Control DP  Control AL vs. Jetlag AL  Control AL vs. Jetlag DP  Control AL vs. Jetlag OP  Control DP vs. Jetlag AL  Control DP vs. Jetlag DP  Control DP vs. Jetlag OP  Jetlag AL vs. Jetlag DP  Jetlag AL vs. Jetlag OP  Jetlag DP vs. Jetlag OP  Day 165  Control AL vs. Control DP  Control AL vs. Jetlag AL  Control AL vs. Jetlag DP  Control AL vs. Jetlag OP  Control DP vs. Jetlag AL  Control DP vs. Jetlag DP  Control DP vs. Jetlag OP  Jetlag AL vs. Jetlag DP  Jetlag AL vs. Jetlag OP  Jetlag DP vs. Jetlag OP  Day 166  Control AL vs. Control DP  Control AL vs. Jetlag AL  Control AL vs. Jetlag DP  Control AL vs. Jetlag OP  Control DP vs. Jetlag AL  Control DP vs. Jetlag DP  Control DP vs. Jetlag OP  Jetlag AL vs. Jetlag DP  Jetlag AL vs. Jetlag OP  Jetlag DP vs. Jetlag OP  Day 167  Control AL vs. Control DP  Control AL vs. Jetlag AL  Control AL vs. Jetlag DP  Control AL vs. Jetlag OP  Control DP vs. Jetlag AL  Control DP vs. Jetlag DP  Control DP vs. Jetlag OP  Jetlag AL vs. Jetlag DP  Jetlag AL vs. Jetlag OP  Jetlag DP vs. Jetlag OP  Day 168  Control AL vs. Control DP  Control AL vs. Jetlag AL  Control AL vs. Jetlag DP  Control AL vs. Jetlag OP  Control DP vs. Jetlag AL  Control DP vs. Jetlag DP  Control DP vs. Jetlag OP  Jetlag AL vs. Jetlag DP  Jetlag AL vs. Jetlag OP  Jetlag DP vs. Jetlag OP  Day 169  Control AL vs. Control DP  Control AL vs. Jetlag AL  Control AL vs. Jetlag DP  Control AL vs. Jetlag OP  Control DP vs. Jetlag AL  Control DP vs. Jetlag DP  Control DP vs. Jetlag OP  Jetlag AL vs. Jetlag DP  Jetlag AL vs. Jetlag OP  Jetlag DP vs. Jetlag OP | Day 163  0.9976  0.8665  >0.9999  >0.9999  0.3505  >0.9999  >0.9999  0.6344  0.5277  >0.9999  Day 164  >0.9999  0.2480  0.9314  0.9995  0.1362  0.9882  0.9898  **0.0107**  0.6761  0.5176  Day 165  >0.9999  **0.0124**  0.9841  0.9296  **0.0051**  0.9990  0.7822  **0.0005**  0.2757  0.2836  Day 166  0.7320  0.7584  0.9936  >0.9999  **0.0342**  0.9987  0.4451  0.2005  0.9400  0.9222  Day 167  0.9864  0.7144  0.9914  >0.9999  0.1420  >0.9999  0.9996  0.1616  0.4740  0.9998  Day 168  >0.9999  >0.9999  0.8152  0.9995  0.9771  0.9786  >0.9999  0.4199  0.9465  0.9933  Day 169  >0.9999  >0.9999  0.9975  >0.9999  >0.9999  >0.9999  >0.9999  0.9921  >0.9999  0.9956 |
| Cumulative ambulatory locomotor activity | Group F (4, 25) = 0.5728, p = 0.6848 | n.a. | n.a. |
| Cumulative periods of behavioral quiescence | Group F (4, 18) = 2.963, **p = 0.0482** | Control AL vs. Control DP  Control AL vs. Jetlag AL  Control AL vs. Jetlag DP  Control AL vs. Jetlag OP  Control DP vs. Jetlag AL  Control DP vs. Jetlag DP  Control DP vs. Jetlag OP  Jetlag AL vs. Jetlag DP  Jetlag AL vs. Jetlag OP  Jetlag DP vs. Jetlag OP | 0.9893  >0.9999  0.2964  0.9960  0.8082  **0.0358**  0.5664  0.5127  >0.9999  0.7478 |
| Cumulative behavioral quiescence in light phase | Group F (4, 18) = 10.12, **p = 0.0002** | Control AL vs. Control DP  Control AL vs. Jetlag AL  Control AL vs. Jetlag DP  Control AL vs. Jetlag OP  Control DP vs. Jetlag AL  Control DP vs. Jetlag DP  Control DP vs. Jetlag OP  Jetlag AL vs. Jetlag DP  Jetlag AL vs. Jetlag OP  Jetlag DP vs. Jetlag OP | **0.0055**  0.9687  0.7455  0.9998  **0.0003**  0.1999  **0.0009**  0.1203  0.9998  0.3212 |
| Cumulative behavioral quiescence in dark phase | Group F (4, 18) = 9.196, **p = 0.0003** | Control AL vs. Control DP  Control AL vs. Jetlag AL  Control AL vs. Jetlag DP  Control AL vs. Jetlag OP  Control DP vs. Jetlag AL  Control DP vs. Jetlag DP  Control DP vs. Jetlag OP  Jetlag AL vs. Jetlag DP  Jetlag AL vs. Jetlag OP  Jetlag DP vs. Jetlag OP | 0.0593  0.9999  **0.0061**  >0.9999  **0.0113**  0.9200  0.0900  **0.0011**  0.9869  **0.0083** |
| Bodyweight | group F (4, 133) = 0.314, p = 0.8682  time F (13, 1729) = 193.4, **p < 0.0001**  time x group F (52, 1729) = 2.977, **p < 0.0001** | Week 1  Control AL vs. Control DP  Control AL vs. Jetlag AL  Control AL vs. Jetlag DP  Control AL vs. Jetlag OP  Control DP vs. Jetlag AL  Control DP vs. Jetlag DP  Control DP vs. Jetlag OP  Jetlag AL vs. Jetlag DP  Jetlag AL vs. Jetlag OP  Jetlag DP vs. Jetlag OP  Week 3  Control AL vs. Control DP  Control AL vs. Jetlag AL  Control AL vs. Jetlag DP  Control AL vs. Jetlag OP  Control DP vs. Jetlag AL  Control DP vs. Jetlag DP  Control DP vs. Jetlag OP  Jetlag AL vs. Jetlag DP  Jetlag AL vs. Jetlag OP  Jetlag DP vs. Jetlag OP  Week 5  Control AL vs. Control DP  Control AL vs. Jetlag AL  Control AL vs. Jetlag DP  Control AL vs. Jetlag OP  Control DP vs. Jetlag AL  Control DP vs. Jetlag DP  Control DP vs. Jetlag OP  Jetlag AL vs. Jetlag DP  Jetlag AL vs. Jetlag OP  Jetlag DP vs. Jetlag OP  Week 7  Control AL vs. Control DP  Control AL vs. Jetlag AL  Control AL vs. Jetlag DP  Control AL vs. Jetlag OP  Control DP vs. Jetlag AL  Control DP vs. Jetlag DP  Control DP vs. Jetlag OP  Jetlag AL vs. Jetlag DP  Jetlag AL vs. Jetlag OP  Jetlag DP vs. Jetlag OP  Week 9  Control AL vs. Control DP  Control AL vs. Jetlag AL  Control AL vs. Jetlag DP  Control AL vs. Jetlag OP  Control DP vs. Jetlag AL  Control DP vs. Jetlag DP  Control DP vs. Jetlag OP  Jetlag AL vs. Jetlag DP  Jetlag AL vs. Jetlag OP  Jetlag DP vs. Jetlag OP  Week 11  Control AL vs. Control DP  Control AL vs. Jetlag AL  Control AL vs. Jetlag DP  Control AL vs. Jetlag OP  Control DP vs. Jetlag AL  Control DP vs. Jetlag DP  Control DP vs. Jetlag OP  Jetlag AL vs. Jetlag DP  Jetlag AL vs. Jetlag OP  Jetlag DP vs. Jetlag OP  Week 13  Control AL vs. Control DP  Control AL vs. Jetlag AL  Control AL vs. Jetlag DP  Control AL vs. Jetlag OP  Control DP vs. Jetlag AL  Control DP vs. Jetlag DP  Control DP vs. Jetlag OP  Jetlag AL vs. Jetlag DP  Jetlag AL vs. Jetlag OP  Jetlag DP vs. Jetlag OP  Week 15  Control AL vs. Control DP  Control AL vs. Jetlag AL  Control AL vs. Jetlag DP  Control AL vs. Jetlag OP  Control DP vs. Jetlag AL  Control DP vs. Jetlag DP  Control DP vs. Jetlag OP  Jetlag AL vs. Jetlag DP  Jetlag AL vs. Jetlag OP  Jetlag DP vs. Jetlag OP  Week 17  Control AL vs. Control DP  Control AL vs. Jetlag AL  Control AL vs. Jetlag DP  Control AL vs. Jetlag OP  Control DP vs. Jetlag AL  Control DP vs. Jetlag DP  Control DP vs. Jetlag OP  Jetlag AL vs. Jetlag DP  Jetlag AL vs. Jetlag OP  Jetlag DP vs. Jetlag OP  Week 19  Control AL vs. Control DP  Control AL vs. Jetlag AL  Control AL vs. Jetlag DP  Control AL vs. Jetlag OP  Control DP vs. Jetlag AL  Control DP vs. Jetlag DP  Control DP vs. Jetlag OP  Jetlag AL vs. Jetlag DP  Jetlag AL vs. Jetlag OP  Jetlag DP vs. Jetlag OP  Week 21  Control AL vs. Control DP  Control AL vs. Jetlag AL  Control AL vs. Jetlag DP  Control AL vs. Jetlag OP  Control DP vs. Jetlag AL  Control DP vs. Jetlag DP  Control DP vs. Jetlag OP  Jetlag AL vs. Jetlag DP  Jetlag AL vs. Jetlag OP  Jetlag DP vs. Jetlag OP  Week 23  Control AL vs. Control DP  Control AL vs. Jetlag AL  Control AL vs. Jetlag DP  Control AL vs. Jetlag OP  Control DP vs. Jetlag AL  Control DP vs. Jetlag DP  Control DP vs. Jetlag OP  Jetlag AL vs. Jetlag DP  Jetlag AL vs. Jetlag OP  Jetlag DP vs. Jetlag OP  Week 25  Control AL vs. Control DP  Control AL vs. Jetlag AL  Control AL vs. Jetlag DP  Control AL vs. Jetlag OP  Control DP vs. Jetlag AL  Control DP vs. Jetlag DP  Control DP vs. Jetlag OP  Jetlag AL vs. Jetlag DP  Jetlag AL vs. Jetlag OP  Jetlag DP vs. Jetlag OP  Week 27  Control AL vs. Control DP  Control AL vs. Jetlag AL  Control AL vs. Jetlag DP  Control AL vs. Jetlag OP  Control DP vs. Jetlag AL  Control DP vs. Jetlag DP  Control DP vs. Jetlag OP  Jetlag AL vs. Jetlag DP  Jetlag AL vs. Jetlag OP  Jetlag DP vs. Jetlag OP | Week 1  0.9992  0.9969  0.7619  0.9986  >0.9999  0.2994  >0.9999  0.2302  >0.9999  0.2793  Week 3  >0.9999  >0.9999  0.7262  0.8643  >0.9999  0.8299  0.9353  0.9538  0.9919  >0.9999  Week 5  0.3023  >0.9999  0.9964  0.9935  0.2428  0.9384  0.9122  0.9920  0.9861  >0.9999  Week 7  0.9985  0.9909  >0.9999  0.7619  0.6838  >0.9999  0.9961  0.9374  0.1562  0.9685  Week 9  >0.9999  >0.9999  0.9980  0.5959  0.9891  >0.9999  0.9173  0.9521  0.2837  0.9925  Week 11  >0.9999  >0.9999  0.9903  0.9784  >0.9999  0.9993  0.9980  0.9983  0.9953  >0.9999  Week 13  >0.9999  >0.9999  >0.9999  >0.9999  0.9992  >0.9999  >0.9999  >0.9999  >0.9999  >0.9999  Week 15  0.9886  0.9349  >0.9999  0.9997  >0.9999  0.9701  >0.9999  0.8886  0.9998  0.9977  Week 17  0.5370  >0.9999  >0.9999  0.9801  0.5000  0.7493  0.0573  >0.9999  0.9832  0.9676  Week 19  >0.9999  0.9999  0.9995  >0.9999  0.9739  0.9618  0.9949  >0.9999  >0.9999  >0.9999  Week 21  0.5822  >0.9999  0.9997  0.9998  0.6462  0.9750  0.9431  >0.9999  >0.9999  >0.9999  Week 23  0.9999  0.9708  0.1933  0.8151  >0.9999  0.5165  0.9903  0.8546  >0.9999  0.9829  Week 25  0.5424  0.9803  >0.9999  0.9992  0.9955  0.9315  0.9567  >0.9999  >0.9999  >0.9999  Week 27  >0.9999  >0.9999  >0.9999  0.9999  >0.9999  >0.9999  0.9997  >0.9999  0.9999  0.9987 |
| Cumulative food intake | Group F (4, 18) = 3.767, **p = 0.0214** | Control AL vs. Control DP  Control AL vs. Jetlag AL  Control AL vs. Jetlag DP  Control AL vs. Jetlag OP  Control DP vs. Jetlag AL  Control DP vs. Jetlag DP  Control DP vs. Jetlag OP  Jetlag AL vs. Jetlag DP  Jetlag AL vs. Jetlag OP  Jetlag DP vs. Jetlag OP | 0.3625  0.8936  0.6107  **0.0137**  0.9952  >0.9999  0.7357  0.9995  0.2223  0.8176 |
| Fat mass | Group F (4, 111) = 1.988, p = 0.1012  time F (7, 777) = 115.6 , **p < 0.0001**  time x group F (28, 777) = 2.669, **p < 0.0001** | Week 0  Control AL vs. Control DP  Control AL vs. Jetlag AL  Control AL vs. Jetlag DP  Control AL vs. Jetlag OP  Control DP vs. Jetlag AL  Control DP vs. Jetlag DP  Control DP vs. Jetlag OP  Jetlag AL vs. Jetlag DP  Jetlag AL vs. Jetlag OP  Jetlag DP vs. Jetlag OP  Week 4  Control AL vs. Control DP  Control AL vs. Jetlag AL  Control AL vs. Jetlag DP  Control AL vs. Jetlag OP  Control DP vs. Jetlag AL  Control DP vs. Jetlag DP  Control DP vs. Jetlag OP  Jetlag AL vs. Jetlag DP  Jetlag AL vs. Jetlag OP  Jetlag DP vs. Jetlag OP  Week 8  Control AL vs. Control DP  Control AL vs. Jetlag AL  Control AL vs. Jetlag DP  Control AL vs. Jetlag OP  Control DP vs. Jetlag AL  Control DP vs. Jetlag DP  Control DP vs. Jetlag OP  Jetlag AL vs. Jetlag DP  Jetlag AL vs. Jetlag OP  Jetlag DP vs. Jetlag OP  Week 12  Control AL vs. Control DP  Control AL vs. Jetlag AL  Control AL vs. Jetlag DP  Control AL vs. Jetlag OP  Control DP vs. Jetlag AL  Control DP vs. Jetlag DP  Control DP vs. Jetlag OP  Jetlag AL vs. Jetlag DP  Jetlag AL vs. Jetlag OP  Jetlag DP vs. Jetlag OP  Week 16  Control AL vs. Control DP  Control AL vs. Jetlag AL  Control AL vs. Jetlag DP  Control AL vs. Jetlag OP  Control DP vs. Jetlag AL  Control DP vs. Jetlag DP  Control DP vs. Jetlag OP  Jetlag AL vs. Jetlag DP  Jetlag AL vs. Jetlag OP  Jetlag DP vs. Jetlag OP  Week 20  Control AL vs. Control DP  Control AL vs. Jetlag AL  Control AL vs. Jetlag DP  Control AL vs. Jetlag OP  Control DP vs. Jetlag AL  Control DP vs. Jetlag DP  Control DP vs. Jetlag OP  Jetlag AL vs. Jetlag DP  Jetlag AL vs. Jetlag OP  Jetlag DP vs. Jetlag OP  Week 24  Control AL vs. Control DP  Control AL vs. Jetlag AL  Control AL vs. Jetlag DP  Control AL vs. Jetlag OP  Control DP vs. Jetlag AL  Control DP vs. Jetlag DP  Control DP vs. Jetlag OP  Jetlag AL vs. Jetlag DP  Jetlag AL vs. Jetlag OP  Jetlag DP vs. Jetlag OP  Week 28  Control AL vs. Control DP  Control AL vs. Jetlag AL  Control AL vs. Jetlag DP  Control AL vs. Jetlag OP  Control DP vs. Jetlag AL  Control DP vs. Jetlag DP  Control DP vs. Jetlag OP  Jetlag AL vs. Jetlag DP  Jetlag AL vs. Jetlag OP  Jetlag DP vs. Jetlag OP | Week 0  >0.9999  >0.9999  >0.9999  >0.9999  >0.9999  >0.9999  >0.9999  >0.9999  >0.9999  >0.9999  Week 4  0.8509  >0.9999  0.8787  0.9998  0.9808  >0.9999  0.9961  0.9846  >0.9999  0.9968  Week 8  0.2294  >0.9999  **0.0034**  0.6428  0.2916  0.7635  0.9998  **0.0047**  0.7322  0.3457  Week 12  0.5610  >0.9999  **0.0104**  0.5958  0.7773  0.6590  >0.9999  **0.0241**  0.8064  0.6268  Week 16  0.7460  >0.9999  **0.0120**  0.9765  0.6559  0.5148  >0.9999  **0.0077**  0.9521  0.1977  Week 20  0.6252  0.9987  0.1949  >0.9999  0.1557  0.9982  0.6331  **0.0291**  0.9985  0.1992  Week 24  0.9919  >0.9999  0.6109  0.9935  0.9996  0.9918  >0.9999  0.7988  0.9998  0.9900  Week 28  0.8349  0.4963  0.7615  0.8753  >0.9999  >0.9999  >0.9999  >0.9999  >0.9999  >0.9999 |
| Lean mass | Group F (4, 111) = 7.365, **p < 0.0001**  time F (7, 777) = 137.1, **p < 0.0001**  time x group F (28, 777) = 2.741, **p < 0.0001** | Week 0  Control AL vs. Control DP  Control AL vs. Jetlag AL  Control AL vs. Jetlag DP  Control AL vs. Jetlag OP  Control DP vs. Jetlag AL  Control DP vs. Jetlag DP  Control DP vs. Jetlag OP  Jetlag AL vs. Jetlag DP  Jetlag AL vs. Jetlag OP  Jetlag DP vs. Jetlag OP  Week 4  Control AL vs. Control DP  Control AL vs. Jetlag AL  Control AL vs. Jetlag DP  Control AL vs. Jetlag OP  Control DP vs. Jetlag AL  Control DP vs. Jetlag DP  Control DP vs. Jetlag OP  Jetlag AL vs. Jetlag DP  Jetlag AL vs. Jetlag OP  Jetlag DP vs. Jetlag OP  Week 8  Control AL vs. Control DP  Control AL vs. Jetlag AL  Control AL vs. Jetlag DP  Control AL vs. Jetlag OP  Control DP vs. Jetlag AL  Control DP vs. Jetlag DP  Control DP vs. Jetlag OP  Jetlag AL vs. Jetlag DP  Jetlag AL vs. Jetlag OP  Jetlag DP vs. Jetlag OP  Week 12  Control AL vs. Control DP  Control AL vs. Jetlag AL  Control AL vs. Jetlag DP  Control AL vs. Jetlag OP  Control DP vs. Jetlag AL  Control DP vs. Jetlag DP  Control DP vs. Jetlag OP  Jetlag AL vs. Jetlag DP  Jetlag AL vs. Jetlag OP  Jetlag DP vs. Jetlag OP  Week 16  Control AL vs. Control DP  Control AL vs. Jetlag AL  Control AL vs. Jetlag DP  Control AL vs. Jetlag OP  Control DP vs. Jetlag AL  Control DP vs. Jetlag DP  Control DP vs. Jetlag OP  Jetlag AL vs. Jetlag DP  Jetlag AL vs. Jetlag OP  Jetlag DP vs. Jetlag OP  Week 20  Control AL vs. Control DP  Control AL vs. Jetlag AL  Control AL vs. Jetlag DP  Control AL vs. Jetlag OP  Control DP vs. Jetlag AL  Control DP vs. Jetlag DP  Control DP vs. Jetlag OP  Jetlag AL vs. Jetlag DP  Jetlag AL vs. Jetlag OP  Jetlag DP vs. Jetlag OP  Week 24  Control AL vs. Control DP  Control AL vs. Jetlag AL  Control AL vs. Jetlag DP  Control AL vs. Jetlag OP  Control DP vs. Jetlag AL  Control DP vs. Jetlag DP  Control DP vs. Jetlag OP  Jetlag AL vs. Jetlag DP  Jetlag AL vs. Jetlag OP  Jetlag DP vs. Jetlag OP  Week 28  Control AL vs. Control DP  Control AL vs. Jetlag AL  Control AL vs. Jetlag DP  Control AL vs. Jetlag OP  Control DP vs. Jetlag AL  Control DP vs. Jetlag DP  Control DP vs. Jetlag OP  Jetlag AL vs. Jetlag DP  Jetlag AL vs. Jetlag OP  Jetlag DP vs. Jetlag OP | Week 0  0.8968  >0.9999  >0.9999  0.9949  0.6662  0.9419  >0.9999  >0.9999  0.9362  0.9980  Week 4  0.0178  0.8753  **0.0004**  **0.0013**  0.4912  0.9349  0.9984  0.0352  0.0999  >0.9999  Week 8  0.1186  0.9114  **0.0040**  0.6167  0.8906  0.9270  0.9955  0.1467  >0.9999  0.4006  Week 12  **0.0084**  0.9547  **<0.0001**  **0.0003**  0.2114  0.4840  0.9935  **0.0007**  **0.0180**  0.9681  Week 16  0.0021  0.1189  **<0.0001**  **<0.0001**  0.9016  0.8828  0.9910  0.1190  0.2718  >0.9999  Week 20  0.1424  >0.9999  **0.0013**  0.4753  0.0505  0.7195  0.9998  **0.0003**  0.2308  0.3129  Week 24  0.0554  0.6618  **<0.0001**  **0.0001**  0.9387  0.2086  0.6635  **0.0072**  **0.0488**  0.9978  Week 28  0.3813  0.9698  0.1786  0.0285  0.9832  >0.9999  0.9784  0.8419  0.3949  >0.9999 |
| Energy expenditure | Group F (4, 107) = 21.87, **p < 0.0001**  time F (6, 652) = 139.21, **p < 0.0001**  time x group F (24, 642) = 3.888, **p < 0.0001** | Week 0-4  Control AL vs. Control DP  Control AL vs. Jetlag AL  Control AL vs. Jetlag DP  Control AL vs. Jetlag OP  Control DP vs. Jetlag AL  Control DP vs. Jetlag DP  Control DP vs. Jetlag OP  Jetlag AL vs. Jetlag DP  Jetlag AL vs. Jetlag OP  Jetlag DP vs. Jetlag OP  Week 4-8  Control AL vs. Control DP  Control AL vs. Jetlag AL  Control AL vs. Jetlag DP  Control AL vs. Jetlag OP  Control DP vs. Jetlag AL  Control DP vs. Jetlag DP  Control DP vs. Jetlag OP  Jetlag AL vs. Jetlag DP  Jetlag AL vs. Jetlag OP  Jetlag DP vs. Jetlag OP  Week 8-12  Control AL vs. Control DP  Control AL vs. Jetlag AL  Control AL vs. Jetlag DP  Control AL vs. Jetlag OP  Control DP vs. Jetlag AL  Control DP vs. Jetlag DP  Control DP vs. Jetlag OP  Jetlag AL vs. Jetlag DP  Jetlag AL vs. Jetlag OP  Jetlag DP vs. Jetlag OP  Week 12-16  Control AL vs. Control DP  Control AL vs. Jetlag AL  Control AL vs. Jetlag DP  Control AL vs. Jetlag OP  Control DP vs. Jetlag AL  Control DP vs. Jetlag DP  Control DP vs. Jetlag OP  Jetlag AL vs. Jetlag DP  Jetlag AL vs. Jetlag OP  Jetlag DP vs. Jetlag OP  Week 16-20  Control AL vs. Control DP  Control AL vs. Jetlag AL  Control AL vs. Jetlag DP  Control AL vs. Jetlag OP  Control DP vs. Jetlag AL  Control DP vs. Jetlag DP  Control DP vs. Jetlag OP  Jetlag AL vs. Jetlag DP  Jetlag AL vs. Jetlag OP  Jetlag DP vs. Jetlag OP  Week 20-24  Control AL vs. Control DP  Control AL vs. Jetlag AL  Control AL vs. Jetlag DP  Control AL vs. Jetlag OP  Control DP vs. Jetlag AL  Control DP vs. Jetlag DP  Control DP vs. Jetlag OP  Jetlag AL vs. Jetlag DP  Jetlag AL vs. Jetlag OP  Jetlag DP vs. Jetlag OP  Week 24-28  Control AL vs. Control DP  Control AL vs. Jetlag AL  Control AL vs. Jetlag DP  Control AL vs. Jetlag OP  Control DP vs. Jetlag AL  Control DP vs. Jetlag DP  Control DP vs. Jetlag OP  Jetlag AL vs. Jetlag DP  Jetlag AL vs. Jetlag OP  Jetlag DP vs. Jetlag OP | Week 0-4  0.4949  0.1588  0.6704  **<0.0001**  0.9999  >0.9999  **<0.0001**  >0.9999  **0.0006**  **0.0009**  Week 4-8  **<0.0001**  **0.0002**  **0.0215**  **<0.0001**  0.9997  0.9248  **0.0379**  0.9987  **0.0045**  **0.0019**  Week 8-12  **<0.0001**  **0.0004**  **0.0002**  **<0.0001**  0.9991  >0.9999  **<0.0001**  0.9981  **<0.0001**  **0.0002**  Week 12-16  **0.0002**  0.2040  **0.0010**  **<0.0001**  0.3129  >0.9999  0.0591  0.4091  **<0.0001**  0.2011  Week 16-20  **0.0111**  0.9986  0.1501  **<0.0001**  0.0908  >0.9999  0.1435  0.5168  **<0.0001**  0.0938  Week 20-24  **<0.0001**  0.3898  0.3536  **<0.0001**  **0.0106**  0.1074  **0.0027**  >0.9999  **<0.0001**  **<0.0001**  Week 24-28  **<0.0001**  0.4555  **0.0013**  **<0.0001**  0.0841  >0.9999  0.0929  0.2335  **<0.0001**  0.1762 |
| Free fatty acids | Group F (4, 133) = 25.93, **p < 0.0001**  time F (3, 399) = 6.599, **p = 0.0001**2  time x group F (12, 399) = 7.42, **p < 0.0001** | Week 0  Control AL vs. Control DP  Control AL vs. Jetlag AL  Control AL vs. Jetlag DP  Control AL vs. Jetlag OP  Control DP vs. Jetlag AL  Control DP vs. Jetlag DP  Control DP vs. Jetlag OP  Jetlag AL vs. Jetlag DP  Jetlag AL vs. Jetlag OP  Jetlag DP vs. Jetlag OP  Week 4  Control AL vs. Control DP  Control AL vs. Jetlag AL  Control AL vs. Jetlag DP  Control AL vs. Jetlag OP  Control DP vs. Jetlag AL  Control DP vs. Jetlag DP  Control DP vs. Jetlag OP  Jetlag AL vs. Jetlag DP  Jetlag AL vs. Jetlag OP  Jetlag DP vs. Jetlag OP  Week 12  Control AL vs. Control DP  Control AL vs. Jetlag AL  Control AL vs. Jetlag DP  Control AL vs. Jetlag OP  Control DP vs. Jetlag AL  Control DP vs. Jetlag DP  Control DP vs. Jetlag OP  Jetlag AL vs. Jetlag DP  Jetlag AL vs. Jetlag OP  Jetlag DP vs. Jetlag OP  Week 20  Control AL vs. Control DP  Control AL vs. Jetlag AL  Control AL vs. Jetlag DP  Control AL vs. Jetlag OP  Control DP vs. Jetlag AL  Control DP vs. Jetlag DP  Control DP vs. Jetlag OP  Jetlag AL vs. Jetlag DP  Jetlag AL vs. Jetlag OP  Jetlag DP vs. Jetlag OP | Week 0  **<0.0001**  **<0.0001**  **<0.0001**  **<0.0001**  0.0546  0.9977  0.8185  0.4726  0.8642  0.9997  Week 4  **0.0412**  >0.9999  0.4056  0.8091  0.0546  0.9977  0.8185  0.4726  0.8642  0.9997  Week 12  **0.0005**  0.9932  **<0.0001**  **<0.0001**  **0.0131**  **0.0450**  0.9683  **<0.0001**  **0.0002**  0.5042  Week 20  0.5121  >0.9999  **0.0079**  **<0.0001**  0.7467  0.6386  **0.0280**  **0.0204**  **<0.0001**  0.9369 |
| Total cholesterol | Group F (4, 139) = 12.29, **p < 0.0001**  time F (3, 417) = 80.01, **p < 0.0001**  time x group F (12, 417) = 7.818, **p < 0.0001** | Week 0  Control AL vs. Control DP  Control AL vs. Jetlag AL  Control AL vs. Jetlag DP  Control AL vs. Jetlag OP  Control DP vs. Jetlag AL  Control DP vs. Jetlag DP  Control DP vs. Jetlag OP  Jetlag AL vs. Jetlag DP  Jetlag AL vs. Jetlag OP  Jetlag DP vs. Jetlag OP  Week 4  Control AL vs. Control DP  Control AL vs. Jetlag AL  Control AL vs. Jetlag DP  Control AL vs. Jetlag OP  Control DP vs. Jetlag AL  Control DP vs. Jetlag DP  Control DP vs. Jetlag OP  Jetlag AL vs. Jetlag DP  Jetlag AL vs. Jetlag OP  Jetlag DP vs. Jetlag OP  Week 12  Control AL vs. Control DP  Control AL vs. Jetlag AL  Control AL vs. Jetlag DP  Control AL vs. Jetlag OP  Control DP vs. Jetlag AL  Control DP vs. Jetlag DP  Control DP vs. Jetlag OP  Jetlag AL vs. Jetlag DP  Jetlag AL vs. Jetlag OP  Jetlag DP vs. Jetlag OP  Week 20  Control AL vs. Control DP  Control AL vs. Jetlag AL  Control AL vs. Jetlag DP  Control AL vs. Jetlag OP  Control DP vs. Jetlag AL  Control DP vs. Jetlag DP  Control DP vs. Jetlag OP  Jetlag AL vs. Jetlag DP  Jetlag AL vs. Jetlag OP  Jetlag DP vs. Jetlag OP | Week 0  0.9997  **0.0234**  0.7428  **0.0037**  **0.0030**  0.3014  **0.0004**  0.7452  0.9998  0.3292  Week 4  **0.0031**  0.5723  **<0.0001**  **0.0091**  0.4648  0.8431  >0.9999  **0.0119**  0.6849  0.6703  Week 12  **0.0007**  0.9043  **<0.0001**  **<0.0001**  0.0577  0.5832  0.9918  **<0.0001**  **0.0033**  0.9941  Week 20  **0.0294**  **0.0027**  0.9997  0.5146  **<0.0001**  0.1603  **<0.0001**  **0.0002**  0.5122  0.1522 |
| Triglycerides | Group F (4, 133) = 24.2, **p < 0.0001**  time F (3, 399) = 8.523, **p < 0.0001**  time x group F (12, 399) = 5.894, **p < 0.0001** | Week 0  Control AL vs. Control DP  Control AL vs. Jetlag AL  Control AL vs. Jetlag DP  Control AL vs. Jetlag OP  Control DP vs. Jetlag AL  Control DP vs. Jetlag DP  Control DP vs. Jetlag OP  Jetlag AL vs. Jetlag DP  Jetlag AL vs. Jetlag OP  Jetlag DP vs. Jetlag OP  Week 4  Control AL vs. Control DP  Control AL vs. Jetlag AL  Control AL vs. Jetlag DP  Control AL vs. Jetlag OP  Control DP vs. Jetlag AL  Control DP vs. Jetlag DP  Control DP vs. Jetlag OP  Jetlag AL vs. Jetlag DP  Jetlag AL vs. Jetlag OP  Jetlag DP vs. Jetlag OP  Week 12  Control AL vs. Control DP  Control AL vs. Jetlag AL  Control AL vs. Jetlag DP  Control AL vs. Jetlag OP  Control DP vs. Jetlag AL  Control DP vs. Jetlag DP  Control DP vs. Jetlag OP  Jetlag AL vs. Jetlag DP  Jetlag AL vs. Jetlag OP  Jetlag DP vs. Jetlag OP  Week 20  Control AL vs. Control DP  Control AL vs. Jetlag AL  Control AL vs. Jetlag DP  Control AL vs. Jetlag OP  Control DP vs. Jetlag AL  Control DP vs. Jetlag DP  Control DP vs. Jetlag OP  Jetlag AL vs. Jetlag DP  Jetlag AL vs. Jetlag OP  Jetlag DP vs. Jetlag OP | Week 0  >0.9999  >0.9999  >0.9999  >0.9999  >0.9999  0.9921  0.9946  0.9999  >0.9999  >0.9999  Week 4  **<0.0001**  0.1349  0.2474  **<0.0001**  0.1808  0.1954  0.8594  >0.9999  **0.0027**  **0.0041**  Week 12  0.9782  0.9947  **0.0030**  **<0.0001**  0.5065  **<0.0001**  **0.0001**  **0.0430**  **<0.0001**  **<0.0001**  Week 20  0.9998  0.6997  0.3877  **<0.0001**  0.9759  0.1137  **<0.0001**  **0.0055**  **0.0044**  **<0.0001** |

**Supplemental Table 2** Comparisons of correlation coefficients of daily core body temperature rhythms (p-values). Top**:** Jetlag AL vs. Control AL, middle: Jetlag DP vs. Control DP, bottom: Jetlag OP vs. Control DP. During odd weeks light-dark schedules are reversed and food is available during the light phase in the Jetlag OP group (bottom) (see Methods section for details). **Control AL** = normal light-dark cycle and food available *ad libitum*; **Jetlag AL** = Jetlag and food available *ad libitum;* **Control DP** = normal light-dark cycle and food available during the dark phase; **Jetlag DP** = Jetlag and food available during the dark phase; **Jetlag OP** = Jetlag and food available alternatingly during the dark or light phase. Correlation coefficients were compared by two-sample t-test, and marked grey when significant (p < 0.05)

| **Days after shift** | |  |  |  | **Jetlag AL vs. Control AL** | | |  |  |  |  |  |  |
| --- | --- | --- | --- | --- | --- | --- | --- | --- | --- | --- | --- | --- | --- |
| **Week** | **1** | **2** | **3** | **4** | **7** | **8** | **11** | **12** | **16** | **19** | **20** | **24** | **25** |
| **Day 1** | 0.096 | 0.000 | 0.407 | 0.148 | 0.494 | 0.010 | 0.095 | 0.427 | 0.113 | 0.864 | 0.415 | 0.004 | 0.872 |
| **Day 2** | 0.000 | 0.000 | 0.000 | 0.000 | 0.000 | 0.000 | 0.043 | 0.001 | 0.000 | 0.000 | 0.001 | 0.000 | 0.000 |
| **Day 3** | 0.000 | 0.000 | 0.001 | 0.312 | 0.000 | 0.003 | 0.001 | 0.168 | 0.011 | 0.000 | 0.080 | 0.002 | 0.000 |
| **Day 4** | 0.000 | 0.320 | 0.000 | 0.497 | 0.014 | 0.261 | 0.002 | 0.637 | 0.169 | 0.000 | 0.198 | 0.142 | 0.000 |
| **Day 5** | 0.000 | 0.150 | 0.000 | 0.656 | 0.000 | 0.378 | 0.018 | 0.346 | 0.292 | 0.000 | 0.875 | 0.035 | 0.000 |
| **Day 6** | 0.000 | 0.888 | 0.000 | 0.740 | 0.000 | 0.286 | 0.021 | 0.699 | 0.318 | 0.001 | 0.721 | 0.314 | 0.029 |
| **Day 7** | 0.000 | 0.250 | 0.000 | 0.593 | 0.004 | 0.303 | 0.000 | 0.172 | 0.883 | 0.035 | 0.322 | 0.045 | 0.030 |
|  |  |  |  |  |  |  |  |  |  |  |  |  |  |
| **Days after shift** | |  |  |  | **Jetlag DP vs. Control DP** | | |  |  |  |  |  |  |
| **Week** | **1** | **2** | **3** | **4** | **7** | **8** | **11** | **12** | **16** | **19** | **20** | **24** | **25** |
| **Day 1** | 0.010 | 0.000 | 0.000 | 0.054 | 0.032 | 0.004 | 0.006 | 0.026 | 0.553 | 0.953 | 0.197 | 0.001 | 0.090 |
| **Day 2** | 0.017 | 0.426 | 0.000 | 0.943 | 0.000 | 0.004 | 0.000 | 0.000 | 0.002 | 0.000 | 0.001 | 0.562 | 0.000 |
| **Day 3** | 0.000 | 0.120 | 0.010 | 0.837 | 0.000 | 0.150 | 0.000 | 0.713 | 0.026 | 0.000 | 0.557 | 0.191 | 0.000 |
| **Day 4** | 0.078 | 0.065 | 0.000 | 0.868 | 0.000 | 0.818 | 0.000 | 0.641 | 0.270 | 0.000 | 0.060 | 0.098 | 0.000 |
| **Day 5** | 0.000 | 0.557 | 0.000 | 0.884 | 0.000 | 0.483 | 0.000 | 0.254 | 0.441 | 0.000 | 0.801 | 0.537 | 0.000 |
| **Day 6** | 0.000 | 0.352 | 0.000 | 0.424 | 0.000 | 0.283 | 0.000 | 0.074 | 0.240 | 0.000 | 0.134 | 0.829 | 0.000 |
| **Day 7** | 0.004 | 0.536 | 0.000 | 0.343 | 0.000 | 0.266 | 0.000 | 0.067 | 0.214 | 0.000 | 0.343 | 0.788 | 0.000 |
|  |  |  |  |  |  |  |  |  |  |  |  |  |  |
| **Days after shift** | |  |  |  | **Jetlag OP vs. Control DP** | | |  |  |  |  |  |  |
| **Week** | **1** | **2** | **3** | **4** | **7** | **8** | **11** | **12** | **16** | **19** | **20** | **24** | **25** |
| **Day 1** | 0.793 | 0.001 | 0.113 | 0.030 | 0.073 | 0.018 | 0.038 | 0.009 | 0.082 | 0.670 | 0.401 | 0.000 | 0.679 |
| **Day 2** | 0.185 | 0.469 | 0.622 | 0.060 | 0.044 | 0.000 | 0.000 | 0.000 | 0.054 | 0.000 | 0.000 | 0.163 | 0.001 |
| **Day 3** | 0.000 | 0.043 | 0.597 | 0.959 | 0.000 | 0.026 | 0.000 | 0.329 | 0.000 | 0.000 | 0.478 | 0.034 | 0.007 |
| **Day 4** | 0.802 | 0.030 | 0.471 | 0.712 | 0.101 | 0.127 | 0.000 | 0.994 | 0.182 | 0.002 | 0.036 | 0.096 | 0.000 |
| **Day 5** | 0.000 | 0.563 | 0.089 | 0.000 | 0.156 | 0.763 | 0.007 | 0.823 | 0.147 | 0.003 | 0.678 | 0.806 | 0.002 |
| **Day 6** | 0.000 | 0.044 | 0.030 | 0.120 | 0.080 | 0.968 | 0.007 | 0.933 | 0.414 | 0.000 | 0.791 | 0.964 | 0.013 |
| **Day 7** | 0.949 | 0.550 | 0.965 | 0.431 | 0.008 | 0.010 | 0.000 | 0.959 | 0.578 | 0.000 | 0.558 | 0.266 | 0.119 |

**Supplemental Figures**

**
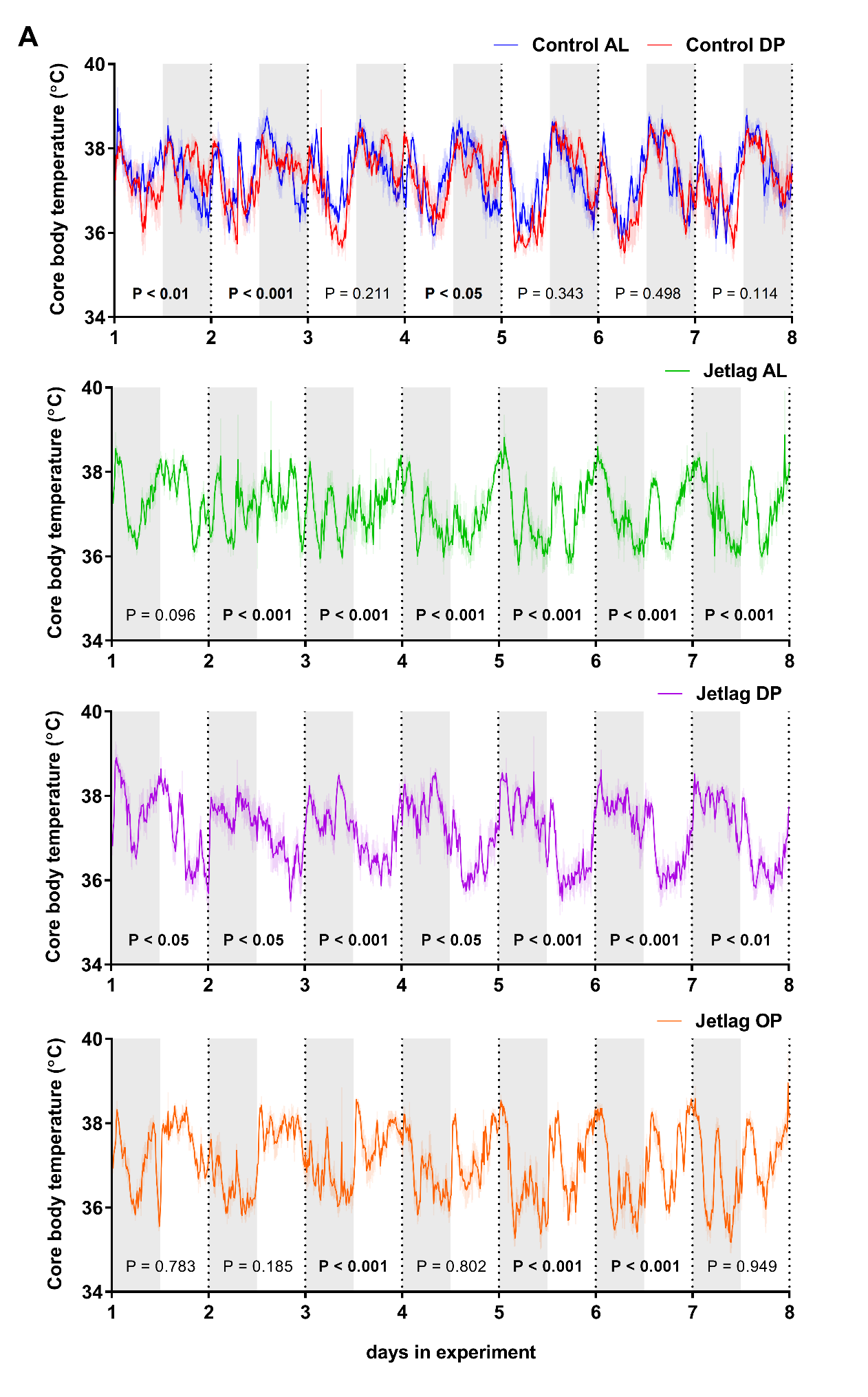
**


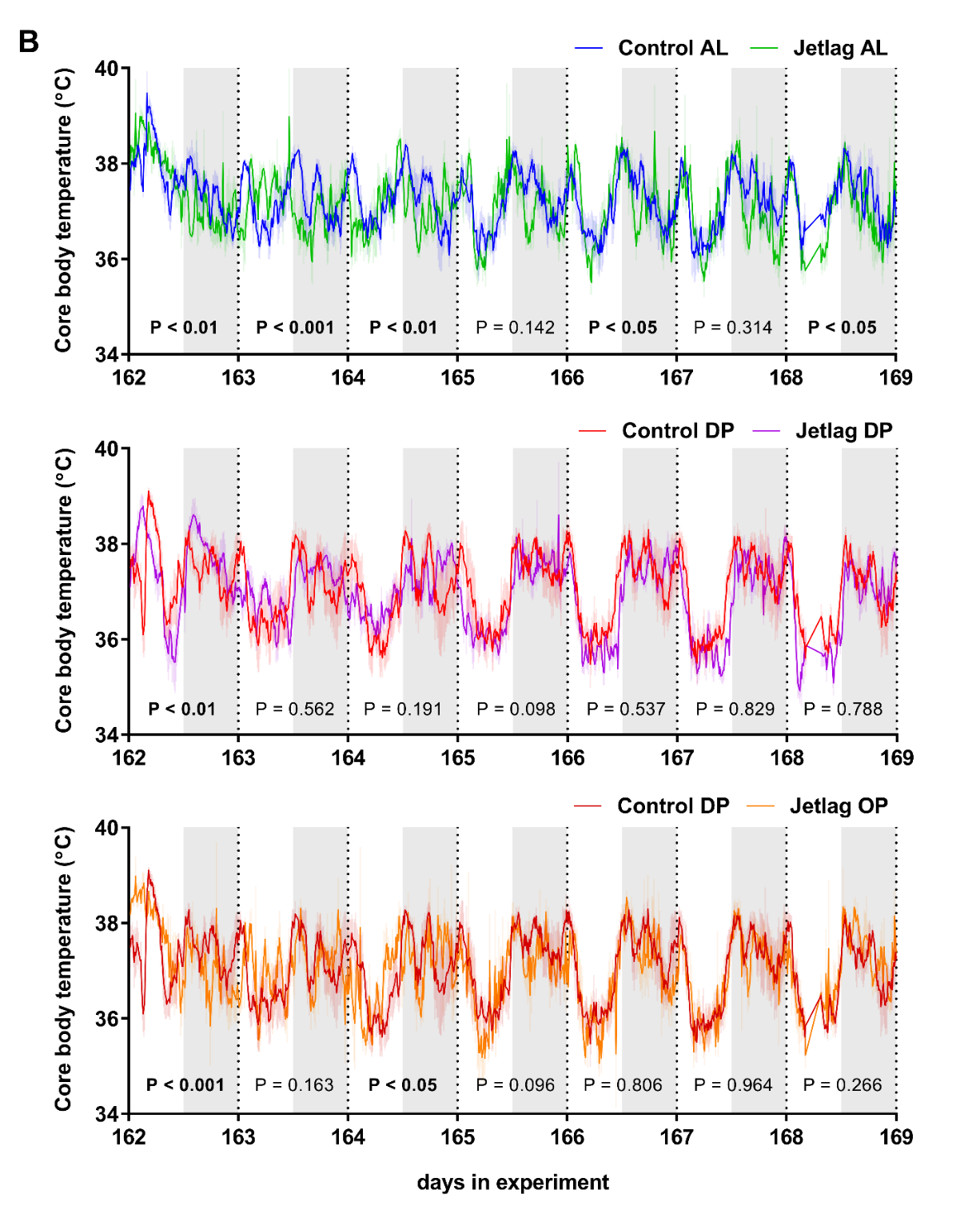


**Supplemental Fig. 1** Daily core body temperature rhythms during week 1 **(A)** and 24 **(B)** of the experiment of the three jetlag groups compared their respective control groups. Data are presented as means ± SEM (as indicated by lighter colored vertical lines), and grey shading indicates the dark phase. P-values represent the comparison of the within-group versus the between-group correlation (as indicated in Methods), and bold p-values indicate a significantly different temperature rhythm in the jetlag group as compared to the respective control group (or the control AL group as compared to the control DP group, for the top graph of panel A). **Control AL** = normal light-dark cycle and food available *ad libitum*; **Jetlag AL** = Jetlag and food available *ad libitum;* **Control DP** = normal light-dark cycle and food available during the dark phase; **Jetlag DP** = Jetlag and food available during the dark phase; **Jetlag OP** = Jetlag and food available alternatingly during the dark or light phase.


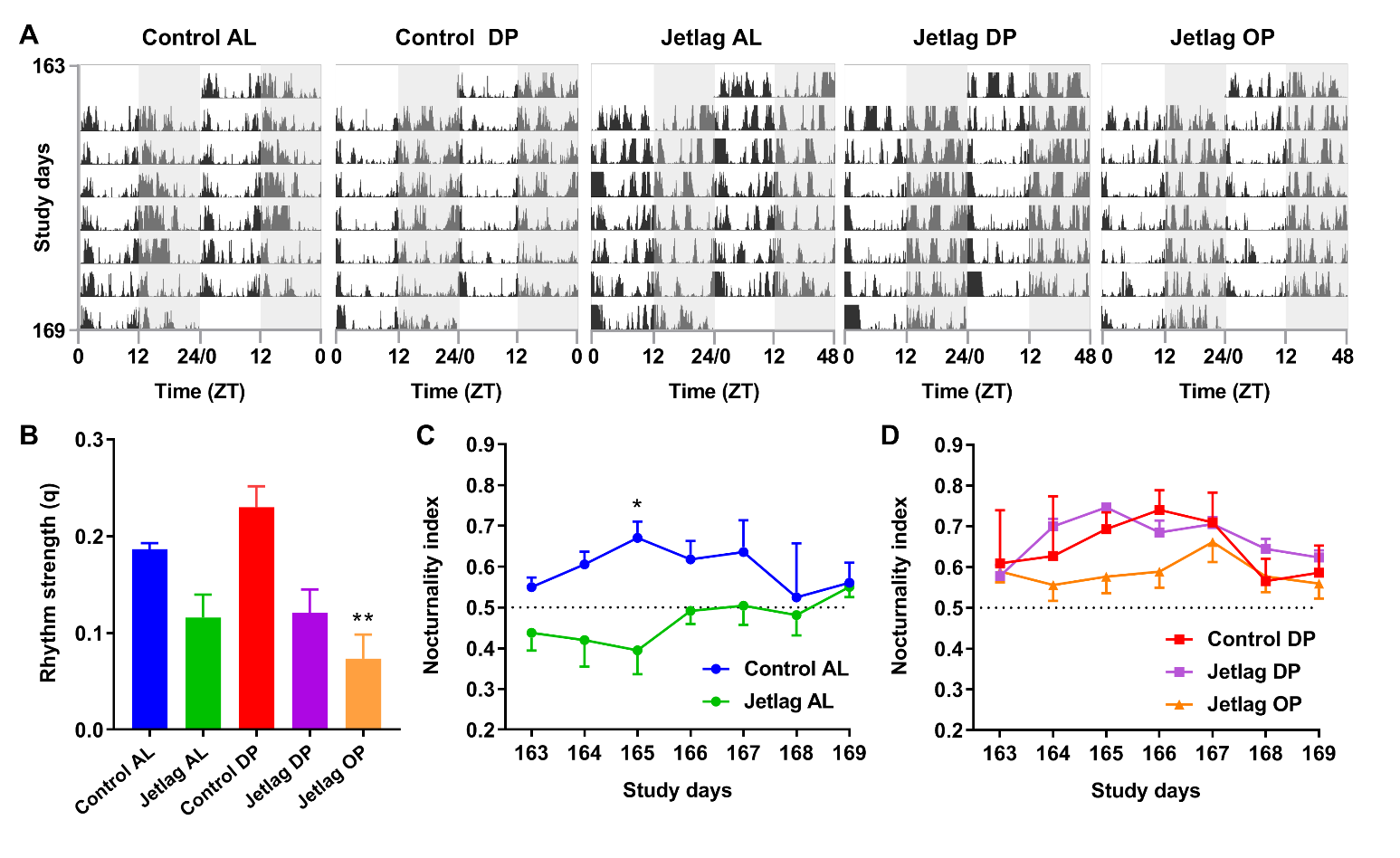


**Supplemental Fig. 2** Representative double-plotted actograms of week 24 of the study are shown, in which grey shading indicates the dark period **(A)**. F-periodogram analysis was performed to calculate rhythm strength (q) of activity over the whole week **(B)**, and the nocturnality index (activity in the dark versus light phase) was calculated to evaluate rhythm strength per day **(C, D)**. **Control AL** = normal light-dark cycle and food available *ad libitum*; **Jetlag AL** = Jetlag and food available *ad libitum;* **Control DP** = normal light-dark cycle and food available during the dark phase; **Jetlag DP** = Jetlag and food available during the dark phase; **Jetlag OP** = Jetlag and food available alternatingly during the dark or light phase. * p < 0.05, ** p < 0.01 vs. control AL. Statistical differences between other groups are not indicated, please see Supplemental Table 1.

**
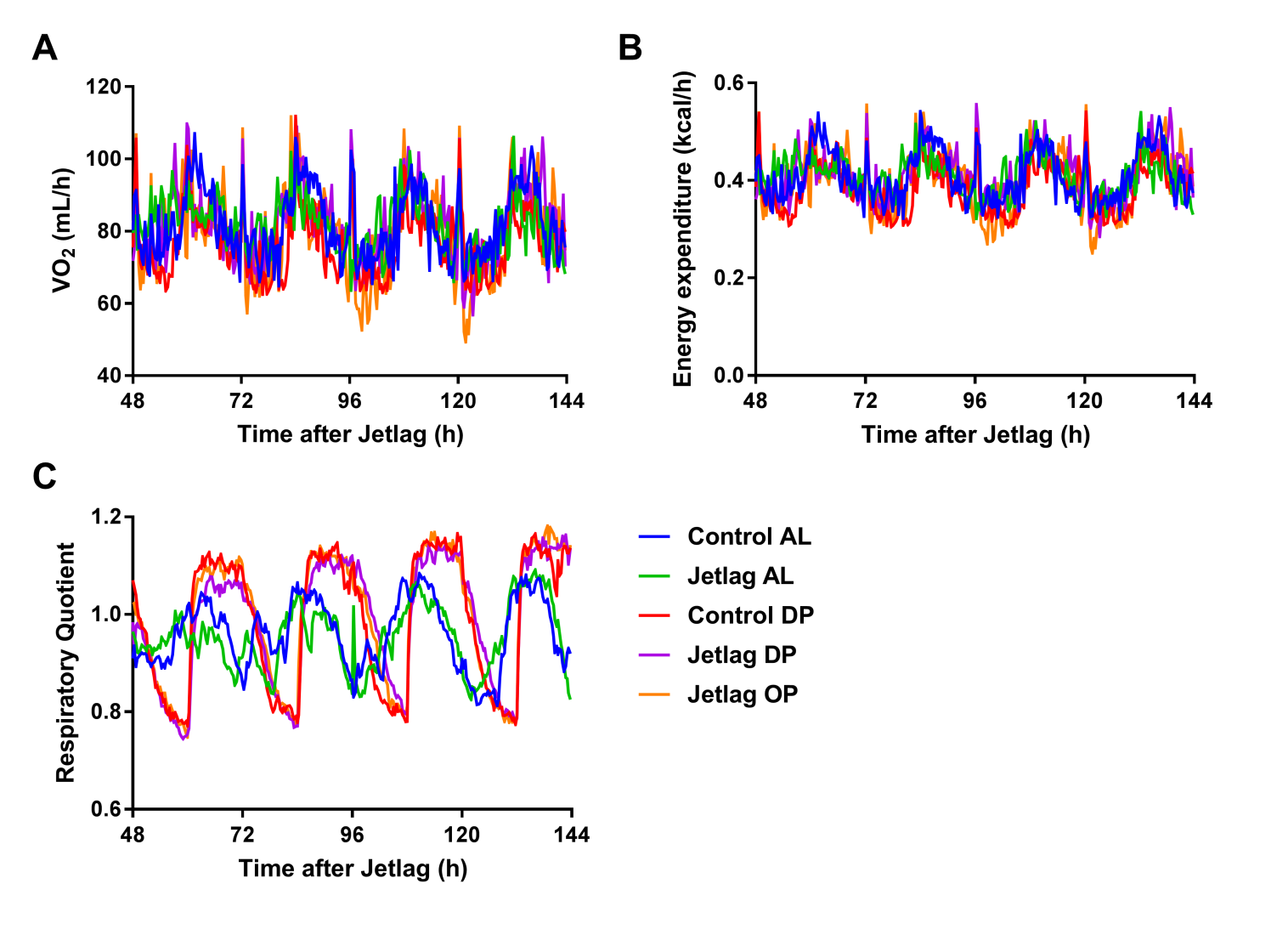
Supplemental Fig.** **3** Daily patterns of oxygen consumption (VO_2_) **(A)**, energy expenditure **(B),** and the respiratory quotient **(C).** **Control AL** = normal light-dark cycle and food available *ad libitum*; **Jetlag AL** = Jetlag and food available *ad libitum;* **Control DP** = normal light-dark cycle and food available during the dark phase; **Jetlag DP** = Jetlag and food available during the dark phase; **Jetlag OP** = Jetlag and food available alternatingly during the dark or light phase.

**
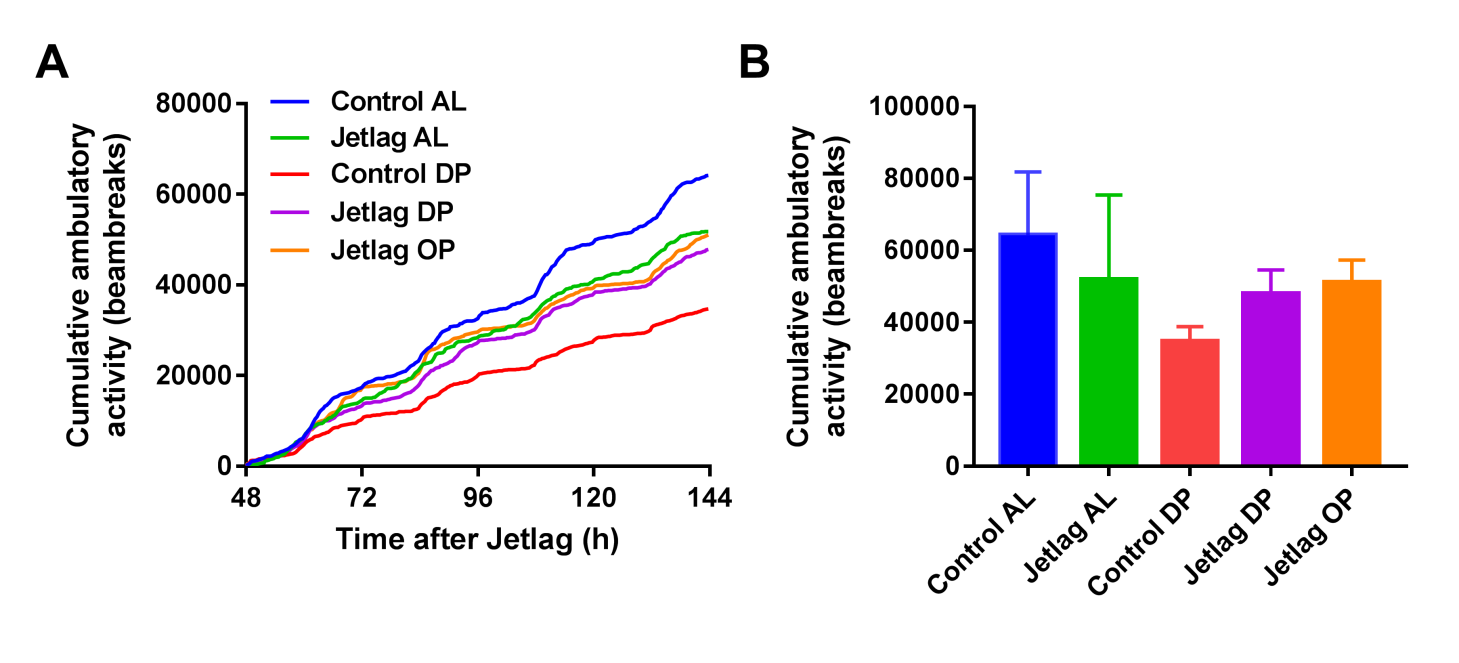
Supplemental Fig. 4** Cumulative ambulatory activity during period of data collection in metabolic cages, after 27 weeks of exposure from day 3 to day 6 after shift **(A)** and total cumulative activity during this period **(B).** **Control AL** = normal light-dark cycle and food available *ad libitum*; **Jetlag AL** = Jetlag and food available *ad libitum;* **Control DP** = normal light-dark cycle and food available during the dark phase; **Jetlag DP** = Jetlag and food available during the dark phase; **Jetlag OP** = Jetlag and food available alternatingly during the dark or light phase.


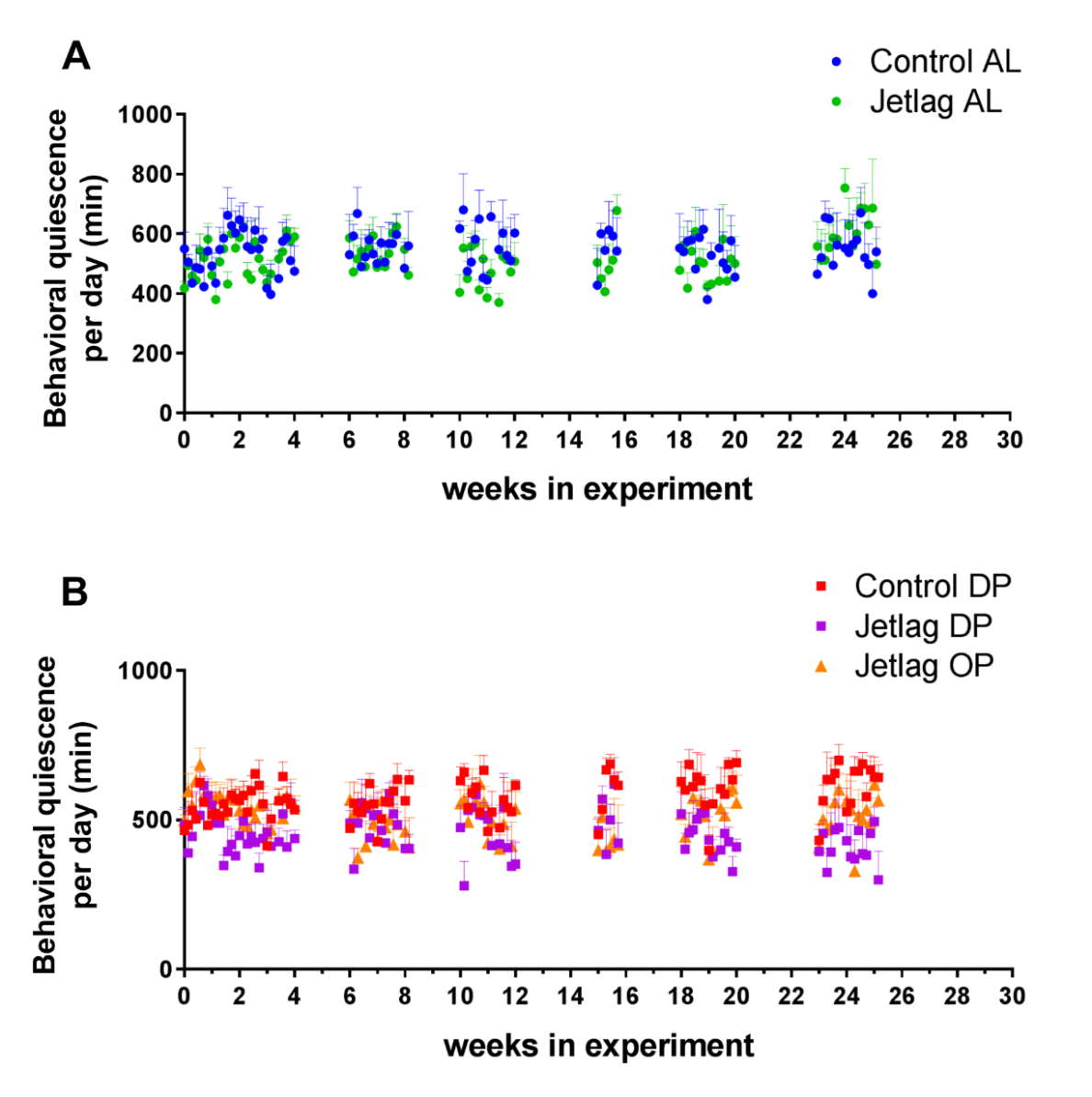


**Supplemental Fig. 5** Cumulative behavioral quiescence (or predicted sleep) in the *ad libitum*-fed mice (**A**) and mice fed at a restricted time (**B**) intermittently measured throughout the study. **Control AL** = normal light-dark cycle and food available *ad libitum*; **Jetlag AL** = Jetlag and food available *ad libitum;* **Control DP** = normal light-dark cycle and food available during the dark phase; **Jetlag DP** = Jetlag and food available during the dark phase; **Jetlag OP** = Jetlag and food available alternatingly during the dark or light phase.
